# Pregnancy induces transient brain and epigenetic aging followed by postpartum rejuvenation

**DOI:** 10.21203/rs.3.rs-10450590/v1

**Published:** 2026-08-13

**Authors:** Susana Carmona, Magdalena Martínez-García, Daniel Martín de Blas, María Paternina-Die, Noelia García Acebes, Hannah Grotzinger, Cristina Ballesteros, Gonzalo López-Montoya, Camila Servín-Barthet, Clara Pretus, Laura Pritschet, Manuel Desco, Elizabeth Chrastil, Hung Phan, Kieran O'Donnell, Emily Jacobs, Oscar Vilarroya

## Abstract

Does pregnancy age or rejuvenate the brain? Although some studies have examined changes in brain aging following childbirth and decades later, the full trajectory of brain aging across gestation and the postpartum period remains uncharacterized. Here, we leverage two unique longitudinal brain imaging datasets to map brain-age trajectories in mothers across their first pregnancy and postpartum. The first one—the largest maternal brain database to date—includes up to 340 gestational mothers scanned at six time points from pre-conception through 16 months postpartum. The second dataset consists of a dense-sampling study of three women scanned up to 27 time points from pre-conception, through pregnancy and up to 2 years postpartum. Hierarchical generalized additive models of T1w anatomical images revealed that brain age gap estimates (signed difference between predicted brain age and chronological age) increased by ~3 years during pregnancy and decreased throughout the postpartum period. By 16 months postpartum, the brain appears rejuvenated, reflected by a negative brain age gap relative to pre-conception levels. These changes were specific to gestational mothers, with no comparable trajectories observed in non-gestational mothers or nulliparous controls, and were robust across four independent brain-age prediction algorithms and replicated across two unique datasets. Additionally, in one densely-sampled participant, brain aging estimates tracked closely with multiple DNA methylation-based epigenetic clocks, signaling concordance between brain and peripheral aging markers. Our findings support the idea that pregnancy induces a transient, multi-system aging-like process, followed by a postpartum rebound that exceeds pre-conception levels.

## INTRODUCTION

Pregnancy induces profound physiological adaptations that place extraordinary metabolic demands on the body[1], with energy expenditure reaching up to ~2.5 times basal rates, comparable to rates observed during elite endurance performance. Following delivery, this intense metabolic state shifts abruptly as the fetus and placenta exit the maternal body. Due to these transient but intense demands, pregnancy has been proposed as a unique model for studying the biology of aging[2]. Gestation-induced metabolic adaptations influence genome function through epigenetic mechanisms such as DNA methylation (DNAm). Variation in DNAm across the lifespan has enabled the development of so-called "epigenetic clocks", algorithms that estimate biological age using peripheral blood. Studies in mice and humans using DNAm-based epigenetic clocks suggest that pregnancy may transiently accelerate peripheral biological aging, followed by a reversal during the early postpartum period[3–5].

Does the dynamic pattern of biological aging and rejuvenation observed in the peripheral tissues during pregnancy extend to the brain? Pregnancy induces profound neuroanatomical changes in the human brain [6–9]. However, whether pregnancy induces a coordinated, multi-system aging and rejuvenation response remains largely unknown. Akin to biological age, brain age estimates can be derived from Magnetic Resonance Image (MRI) data using machine learning models. Brain age gap (BAG) is the difference between a person's predicted age and their chronological age. A positive BAG indicates that the brain appears older than it is, whereas a negative BAG indicates that the brain appears younger. For example, a BAG of +5 in a 32-year-old suggests a brain age pattern typically observed in a 37-year-old, while a BAG of -5 suggests a pattern more typical of a 27-year-old. Because this study examines how BAG changes longitudinally from pre-conception through pregnancy and postpartum, we use the terms "older" and "younger" in a relative sense: increases in BAG indicate an older-appearing brain relative to the BAG of a previous reference time point, whereas decreases in BAG indicate a younger-appearing brain. A postpartum MRI study implementing the BAG found that women's brains appeared "younger" at 4–6 weeks postpartum compared to shortly after childbirth [10]. Such a restoration effect seems to persist well beyond the early postpartum window. Large scale cross-sectional studies in middle-aged women indicate that parous women exhibit younger-looking brains compared to nulliparous women even decades after birth [11–13]. To date, no studies have mapped the evolution of brain and peripheral aging markers from pre-conception through pregnancy and up to 2 years postpartum. Here, we investigated brain age and epigenetic clock trajectories across the full maternal transition.

### Brain age gap increases during pregnancy and reverts postpartum

We longitudinally tracked the BAG in a dataset of 340 first-time gestational mothers from Spain who underwent longitudinal MRI scanning at six time points spanning pre-conception through 16

months postpartum (Figure 1A). To distinguish brain changes related to pregnancy from those associated to parenting, we also collected longitudinal neuroimaging from a subgroup of female same-sex partners of the gestational mothers, referred to as non-gestational mothers. Additionally, to account for brain changes unrelated to pregnancy or parenting, we included a control group of nulliparous women (women without children and no immediate plans for pregnancy or maternity), who were followed over comparable time intervals. This dataset resulted from the combination of two cohorts of Spanish women (Supplementary Table 1). Data collection time points, sample sizes and demographics are summarized in Figure 1A and Supplementary Tables 1-3. We complemented the main dataset with a dense sampling design, comprising three first-time gestational mothers from the United States. Densely sampled subjects underwent more frequent structural MRI scans, with 14, 22 and 27 scans collected from pre-conception through two years postpartum (Figure 1B). We used Hierarchical Generalized Additive Models (HGAM) to characterize the change in BAGover time and Linear Mixed Effects Models (LMEMs) to test the significance of BAGs at each time point relative to a preceding reference time point, with time points coded sequentially from 1 to 6.

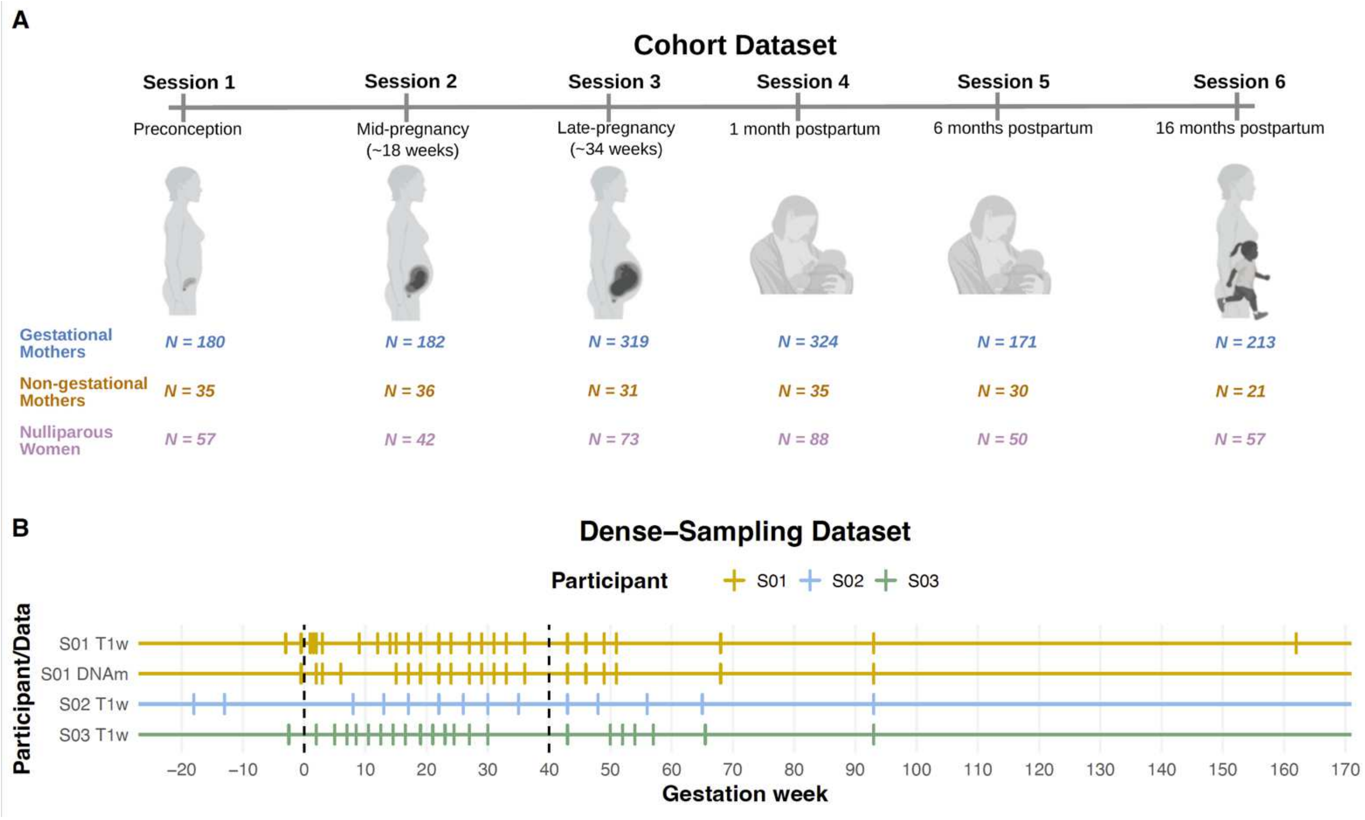

**Figure 1. Overview of the study design: cohort and dense-sampling datasets.** Schematic representation of the two complementary longitudinal designs. A) The cohort dataset includes three groups of participants: gestational mothers, non-gestational mothers and nulliparous women (see sample sizes in the figure), scanned at six consecutive time points through the maternal transition. The total number of women with at least two computed time points equals 340 gestational mothers, 39 non-gestational mothers and 91 nulliparous women. B) The dense-sampling dataset includes repeated, higher-frequency measurements of 3 subjects (S01, S02 and S03). Each vertical line denotes an individual acquisition. Dashed lines at 0 and 40 gestation weeks correspond to standard conception and parturition times, respectively. Note that whereas the cohort dataset collects data at 16 months postpartum, the dense-sampling dataset collects data at 1 year postpartum (S01, S02 and S03), and at 2 years postpartum (S01).

Results indicate a dynamic trajectory of BAG changes across the perinatal period in gestational mothers (Figure 2A and Supplementary Table 4). BAG increased by 2.66 years from pre-conception to late pregnancy (~35 weeks gestation; $estimate_{1\text{-}3}$ = 2.66, $p_{FDR\ 1\text{-}3}$ < 0.001), followed by a BAG decrease of 2.79 years from late pregnancy to 6 months postpartum ($estimate_{3\text{-}5}$ = -2.79, $p_{FDR\ 3\text{-}5}$ < 0.001; temporal dependence: F = 97.84, $p_{FDR}$ = <0.001, Supplementary Figure 1A and Supplementary Table 5). By 6 months postpartum, BAG had returned to pre-conception levels ($estimate_{1\text{-}5}$ = -0.13, $p_{FDR\ 1\text{-}5}$ = 0.699), and by 16 months postpartum, BAG was significantly lower than pre-conception levels; indicating that the brain appeared significantly "younger" relative to the mothers' chronological age ($estimate_{1\text{-}6}$ = -0.50, $p_{FDR\ 1\text{-}6}$ = 0.021) (see also Supplementary Table 5). Findings were replicated in the independent dense-sampling dataset (Figure 2B, Supplementary Figure 1B, Supplementary Table 6 and 7). BAG increased by 3.12 years from pre-conception to late pregnancy (*estimate* = 3.12, $p_{FDR}$ < 0.001), then decreased by 3.18 years between late pregnancy and 6 months postpartum (*estimate* = -3.18, $p_{FDR}$< 0.001). By 1 year postpartum, the brain appeared significantly younger relative to the mothers' chronological age, resulting in a BAG significantly lower than pre-conception (*estimate* = -2.47, $p_{FDR}$ = 0.003). This effect reflects changes in BAG, rather than a reduction in the predicted brain age itself, which increased over time consistent with ongoing chronological aging. All models were adjusted for chronological age, indicating that these effects were independent of normative aging.

No significant longitudinal changes in BAG were observed in non-gestational mothers (HGAM temporal smooth term $p_{FDR}$ = 0.241, all LMEM $p_{FDR}$ > 0.173) or in nulliparous women (HGAM temporal smooth term $p_{FDR}$ = 0.135, all LMEM $p_{FDR}$ > 0.160) (Figure 2C-D, Supplementary Figure 1A, Supplementary Table 4 and 5). Baseline comparisons confirmed no pre-existing differences in BAG between gestational and non-gestational mothers (p = 0.933, Supplementary Table 5), or between gestational mothers and nulliparous women (p = 0.215, Supplementary Table 5). Additional LMEM analyses revealed no significant influence of delivery mode (vaginal delivery, emergency cesarean (c-)section and scheduled c-section) on the observed BAG trajectories (Supplementary Table 8). A BAG difference between vaginal delivery and scheduled c-section were detected from preconception to 16 months postpartum (p=0.024, $p_{FDR}$=0.061), however it did not survive multiple corrections. Overall, findings suggest that the observed brain age changes were specific to gestational mothers and primarily driven by pregnancy-related factors. We used *brainageR* to compute BAG because it demonstrated the highest test–retest reliability among six publicly available brain age prediction algorithms, while also showing high accuracy in predicting chronological age [14]. To test the robustness of the results, we repeated HGAM and LMEM analyses using three independent brain age prediction models—*Pyment*, *DeepBrainNet*, and *BrainAGE*. All three models consistently reproduced the characteristic pattern of brain aging during pregnancy followed by postpartum brain restoration. While the overall trajectory of the cohort dataset was consistent across models (Supplementary Figure 2 and Supplementary Tables 9, 10, 11, 12, 13 and 14), some differences emerged in the extent of postpartum brain restoration relative to pre-conception levels. By 16 months postpartum, all models detected a younger-looking brain ($estimates_{1\text{-}6}$ <0; Supplementary Tables 12, 13 and 14). This finding reached statistical significance in the *BrainAGE* ($\beta$ = $-0.861_{1\text{-}6}$, $p_{FDR\ 1\text{-}6}$ < 0.001), and *DeepBrainNet* ($\beta$ = $-1.094_{1\text{-}6}$, $p_{FDR\ 1\text{-}6}$ = 0.001) models, whereas it remained non-significant in the *Pyment* ($\beta$ = -

$0.218_{1\text{-}6}$, $p_{FDR\ 1\text{-}6} = 0.514$) model. Despite the smaller sample size of the dense-sampling dataset, all three models also replicated the pattern of significant BAG increases during pregnancy followed by decreases during the postpartum (Supplementary Figure 3 and Supplementary Tables 15 and 16). After the first postpartum year, all models estimated a younger-looking brain (all estimates< 0), although these effects did not reach statistical significance, likely due to a limited sample size (Supplementary Table 16).

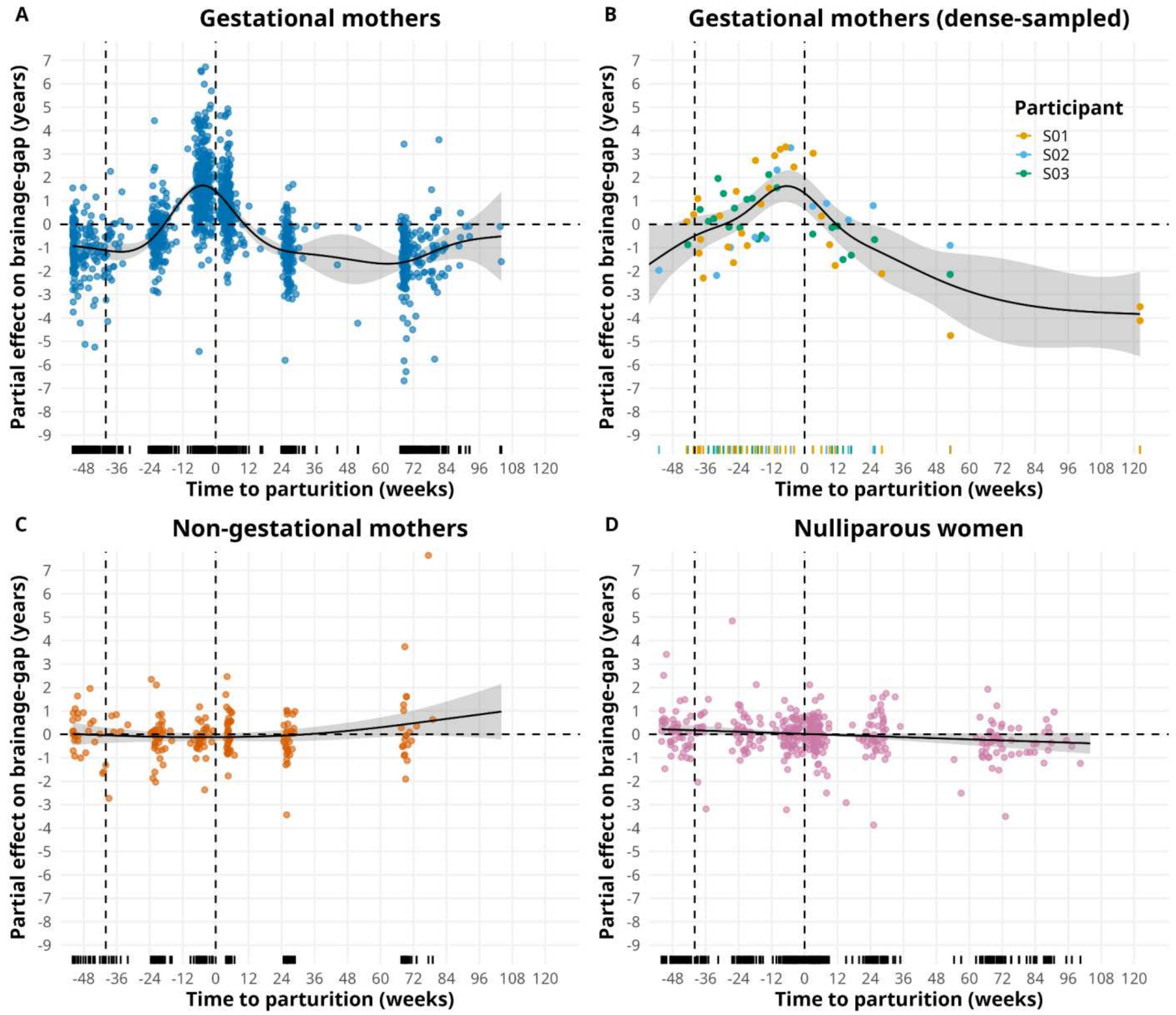

**Figure 2.** Brain age gap trajectories estimated using Hierarchical Generalized Additive Models (HGAM) with the *BrainageR* algorithm. Trajectories are shown for: **A)** first-time gestational mothers in the cohort dataset; **B)** first-time gestational mothers in the dense-sampling dataset, where each color represents a different subject (S); **C)** non-gestational mothers in the cohort dataset; and **D)** nulliparous women in the cohort dataset. Dashed vertical lines indicate nominal times for conception (x=-40) and parturition (x=0). Gray shadows indicate pointwise 95% confidence intervals.

### Coupling of Brain and Epigenetic Age Clocks During Pregnancy and Postpartum

For one gestational mother of the dense-sampling dataset (S01; 27 MRI scans throughout the maternal transition; Figure 3A), we integrated brain imaging data with blood-derived DNAm based estimates of biological age to examine concordance of pregnancy-induced aging trajectories across systems. Using 24 whole blood samples collected at 20 time points (19 coincident with MRI scans), we derived DNAm and estimated biological age using principal component-based epigenetic clocks (PCHorvath1, PCHorvath2, PCPhenoAge, PCGrimAge), which are more robust to technical variation and more reliable for longitudinal studies than conventional (CpG) clocks. We also applied an updated version of the GrimAge estimator (GrimAge2[15]) and a biomarker of the pace of biological aging (DunedinPACE[16]). We define biological age gap as the signed difference between the estimated biological age minus chronological age, while the pace of biological aging is expressed in biological years per chronological year. We used generalized additive models (GAM) to characterize how biological age gap changed over time, and Pearson correlation to test associations between the biological age gap, biological aging pace, and BAG.

We found a positive association between pregnancy stage and all DNAm-based biomarkers of aging, followed by a reversal from parturition to approximately 4 months postpartum (see Figure 3B-C for PCGrimAge and DunedinPACE epigenetic clocks, and Supplementary Figure 4 and Supplementary Table 17 for the remaining clocks). Predicted biological age increased by 4.5 years from pre-conception to parturition according to the PCGrimAge epigenetic clock, and by 0.28 units according to the DuneinPACE biomarker of aging pace. We also observed a 5.5 years "reversal" of biological aging across all epigenetic biomarkers using PCGrimAge and 0.27 units using DunedinPACE. This reversal began near parturition and continued through approximately 4 months postpartum, with effects sustained at 1 year postpartum. This aging trajectory resembles that observed in brain age.

All epigenetic clocks correlated with BAG trajectories obtained with *brainageR* , suggesting that brain and biological aging covary throughout pregnancy and postpartum (see Figure 3 D-E for *brainageR* model, and *PCGrimAge* and *DunedinPACE* epigenetic clocks, Supplementary Figure 4 for *brainageR* model and the remaining epigenetic clocks, and Supplementary Table 18 for the remaining brain age models and epigenetic clocks correlations).

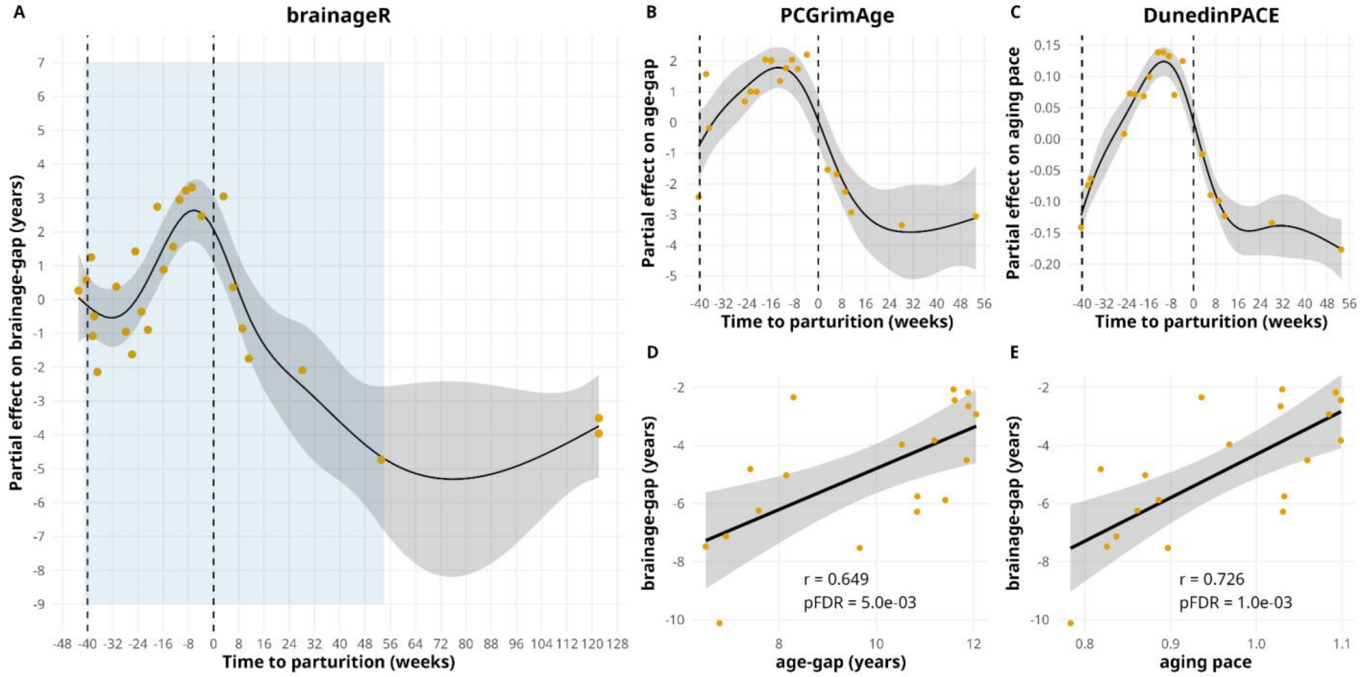

**Figure 3. Coupling between brain and biological aging markers in a densely sampled gestational mother.** Panels show: **A)** Brain aging trajectory; **B)** PCGrimAge trajectory; **C)**

DunedinPACE trajectory; **D)** Correlation between brain age gap (BAG) and PCGrimAge; **E)** Correlation between BAG and DunedinPACE. Blue shaded area in panel A corresponds to the time frame in which epigenetic aging data is available. From A to C, each point represents the partial estimates of the adjusted GAM model. Dashed lines indicating at x=0 and x=40 correspond to nominal conception and childbirth times, respectively. Gray shadows represent the 95% confidence interval, r is the Pearson correlation coefficient and pFDR is the associated family-wise rate corrected p-value. Model adjusted: metric ~ s(time to parturition).

## DISCUSSION

This study reveals pregnancy-related changes in brain aging, characterized by a transient increase during pregnancy followed by a postpartum rebound that exceeds pre-conception levels. By mapping the complete trajectory of BAG from pre-conception to 16 months postpartum, we identify a postpartum *rejuvenation* pattern in gestational mothers that exceeds the transient increase in brain age observed across pregnancy. Importantly, by 16 months postpartum, BAG is reduced approximately 0.5 years compared to pre-conception, and this is not observed in nulliparous controls or non-gestational mothers, supporting a pregnancy-related effect rather than measurement variability.

The observation of a "younger-looking" brain at late postpartum aligns with prior cross-sectional evidence linking parity to long-lasting neural changes. In retrospective analyses of approximately 12,000 middle-aged women from the UK Biobank, parous women exhibited less evidence of brain aging, based on structural brain patterns, than nulliparous women, with effects most pronounced among multiparous mothers[11–13]. Our prospective longitudinal design demonstrates that pregnancy itself actively shapes brain aging trajectories and that the rejuvenation effect is already detectable by approximately 16 months postpartum. Longitudinal studies spanning multiple pregnancies will be needed to determine whether repeated childbirths exert cumulative effects on maternal brain aging trajectories.

Brain age algorithms estimate "brain age" from structural T1-weighted MRI scans using machine learning models that integrate information from gray matter, white matter, and cerebrospinal fluid. Among these features, cortical thickness declines steadily with chronological aging [17]. In previous work, we reported robust cortical thinning during pregnancy that did not fully recover postpartum at 2 and 6 years postpartum [6,7,18]. It is possible that changes in cortical thickness contribute to the observed changes in brain age. However, the present analyses show that BAG estimates not only return to baseline after pregnancy but fall below pre-conception levels. This suggests that additional neuroanatomical processes could be contributing to the observed brain age changes, particularly those underlying the postpartum restoration effect.

To further examine whether these neural trajectories reflect broader biological aging dynamics, we integrated longitudinal DNAm epigenetic clocks from a densely sampled individual across the same reproductive time window. Brain age has been modestly linked to DNAm estimates of biological age in cross-sectional analyses[19]. Results showed that BAG trajectories during pregnancy and postpartum were mirrored by multiple epigenetic aging markers. Specifically, biological age and aging pace increased across pregnancy, followed by a reversal from childbirth to approximately four months postpartum across all examined clocks. The progressive reduction in biological age gap across the postpartum period ultimately resulted in a "younger" predicted biological age at 16 months postpartum, tracking with the brain volumetric findings. This temporal pattern aligns with prior murine and human studies tracking DNAm clocks across the perinatal period. For instance, Poganik and colleagues showed that biological age in mice rose during pregnancy but normalized after parturition[4]. Human studies likewise indicate accelerated biological aging across gestation [4,5,20–23], and most recently, Pham et al. (2024) reported a reversal of pregnancy-associated age acceleration by 3 months postpartum[3]. However, these studies captured only discrete phases of the maternal transition: some were restricted to pregnancy alone [20,22], others lacked pre-conception baselines [3,20,22,23], and none extended beyond the early postpartum period (3 months postpartum). By contrast, our dataset delineates for the first time the full trajectory of epigenetic aging from pre-conception through pregnancy and up to 1 year postpartum.

Pregnancy has been proposed as a model of aging due to its profound physiological demands on the maternal body [2]. The perinatal period is marked by heightened vulnerability to cardiovascular complications (e.g., preeclampsia, hemorrhage), metabolic disorders (e.g., gestational diabetes), and infections (e.g., urinary tract infections). Paradoxically, however, parous women have lower rates of all-cause mortality[24] and premature death [25] than nulliparous women, suggesting the physiological 'cost' of pregnancy for many women might be offset by postpartum recovery effects or a better health-related quality of life detected in mothers [26]. Similarly, although motherhood imposes substantial cognitive demands during the peripartum period, sustained engagement with these challenges across the lifespan has been suggested to lead to greater late-life cognitive reserve [27,28]. Analogous to endurance exercise, in which acute metabolic stress may be followed by long-term adaptive benefits, the transient increase in brain and biological aging observed during pregnancy may reflect both an immediate metabolic cost and a longer-term shift toward a more youthful biological profile.

More broadly, these observations align with emerging evidence that biological aging trajectories are non-linear, with specific periods of rapid change [29]. Our results suggest that pregnancy may represent an additional window during which biological aging processes are transiently accelerated, followed by a period of recovery or decelerated aging. It will be crucial to determine whether the magnitude of postpartum recovery predicts risk of age-related illness in women, and whether it is moderated by factors such as nutritional environment and psychosocial adversity, which may serve as new intervention targets to promote women's health.

At the same time, these results raise important questions about how aging algorithms interpret the physiological adaptations of pregnancy. Pregnancy induces physiological states that resemble certain hallmarks of aging, including oxidative stress, inflammation, and vascular remodeling [30]. Aging algorithms may misclassify these dynamic, adaptive changes as accelerated aging, when in fact they could reflect the heightened allostatic load required to sustain pregnancy—that is, the body's coordinated recalibration across multiple systems to maintain stability under extreme physiological demand. Alternatively, pregnancy may transiently increase stochasticity—the degree of random variability or "noise" in biological processes—which has been shown to inflate estimates of biological age in methylome-based models [31]. Such stochastic fluctuations may contribute to the apparent rise in biological age during pregnancy and its subsequent reversal postpartum.

These observations also underscore the urgent need to develop large-scale, female-centered brain databases. Current brain age models are limited by the characteristics of the datasets they were trained on, which almost never include information on pregnancy or parity status. Expanding databases to capture these critical variables is essential for accurately characterizing female brain trajectories across the reproductive lifespan. Moreover, most available evidence is derived from retrospective datasets, such as the UK Biobank, which limits the ability to disentangle pregnancy-specific effects from those related to aging, parenthood, and broader midlife factors. Prospective longitudinal designs, such as the present study, are therefore critical for isolating the unique contributions of pregnancy to brain aging dynamics.

This study offers several unique strengths that advance not only our understanding of maternal brain adaptations but also of biological processes of accelerated aging and rejuvenation. Nonetheless, some limitations should be acknowledged. First, peripheral biological age estimates were available from only one densely sampled participant, limiting generalizability. Larger longitudinal studies combining neuroimaging with epigenetic and hormonal biomarkers are needed to further characterize these findings. Second, future research should consider factors such as fertility treatments, breastfeeding, mental health, sleep, and pregnancy complications, which may influence aging trajectories. Likewise, multi-pregnancy databases are essential to determine whether rejuvenation accumulates or diminishes over time, as prior findings are mixed [32,33]. Finally, studies with sufficient resolution at the cellular level are needed to determine whether the observed postpartum rejuvenation reflects reversal of pregnancy-induced changes or activation of postpartum-specific restorative processes.

Our findings provide longitudinal evidence that pregnancy initiates a dynamic neurobiological trajectory—one that temporarily accelerates apparent brain aging but is followed by sustained recovery, highlighting the perinatal period as a potential window for resilience and rejuvenation.

## METHODS

### Datasets

In this study we use two complementary longitudinal datasets of first-time mothers in their transition to motherhood, a cohort dataset with 340 gestational mothers scanned up to six temporal points, and a dense-sampling dataset with three gestational mothers scanned from 14 up to 27 temporal points. All participants gave written informed consent and the studies that collected the data were approved by the Hospital del Mar Research Institute (Ref: 2017/7450/I), the Hospital Clínic de Barcelona (Ref: HCB/2018/0357), the Hospital Universitari Quirón Dexeus (Ref: 7/2/2017), the Ethics Committee at the Instituto de Investigación Sanitaria del Hospital Gregorio Marañón (ref: 106/19), and the University of California Irvine Human Subjects Committee (ref: 20195448).

### Cohort Dataset

The cohort dataset includes data acquired in two sites in Spain, Barcelona and Madrid, with compatible temporal points and data acquisition protocols. In the Barcelona site, participants were scanned up to six temporal points (sessions): (1) pre-conception, (2) at 18 weeks of pregnancy, (3) at 34 weeks of pregnancy, (4) at one month postpartum, (5) at six months postpartum, and (6) at 16 months postpartum (see Supplementary Table 1). Participants include gestational mothers, non-gestational mothers and a control group of nulliparous women that did not transition to motherhood, scanned at matching time intervals. At each session, a T1-weighted image was acquired on a Philips Ingenia CX MRI scanner with a Head-and-Neck 32-channel coil, located at the Barcelona Beta Brain Research Center, with the following parameters: voxel size, 1mm × 0.75mm × 0.75mm; field of view, 180mm × 240mm × 240mm; echo time, 4.6 ms; repetition time, 9.9/2300 ms; prepulse delay, 900 ms; flip angle, 8º; acceleration factor, 1.9; percent sampling, 78%. In the Madrid site, participants were scanned at three temporal points, with similar timings to sessions 3, 4 and 6 of the Barcelona site (Supplementary Table 1). At each session, a T1-weighted image was acquired on a Siemens Vida XQ with a Head-and-Neck 20 channel coil, located at Hospital Beata Maria Ana, with the following parameters: voxel size, 1mm × 0.9375mm × 0.9375mm; field of view, 176 mm × 240mm × 240mm ; echo time, 4.4 ms; repetition time, 9.8/2300 ms; inversion time, 900 ms; flip angle, 8º; GRAPPA acceleration factor, 2; percent sampling, 80%.

In this dataset we only included participants for which it was possible to compute brain-age data in at least two temporal points, leading to 340 gestational mothers, 37 non-gestational mothers and 91 control nulliparous women. For full demographic, sample sizes and temporal data of this cohort dataset see Supplementary Tables 1, 2 and 3. Additional information about this dataset can be obtained elsewhere (Barcelona: Servin-Barthet et al. 2025; Madrid: Paternina-Die et al. 2024).

**Dense-sampling dataset**

The dense-sampling dataset contains data from three healthy primiparous women acquired in California, US. All three self-identify as White/Caucasian. Participant S01 (conceived via in vitro fertilization (IVF) at 38 years old and gave birth vaginally at 39 gestational weeks) underwent 27 MRI sessions from pre-conception to two years postpartum (Figure 1B) using a Siemens 3T Prisma MRI scanner. First 6 sessions and last session took place at UCSB Brain Imaging Center (BIC), while the remaining 20 sessions took place at UCI Facility for Imaging and Brain Research (FIBRE). At each session, T1-weighted images were acquired with the following acquisition parameters: voxel size, 0.8mm × 0.8mm × 0.8 mm; field of view, 166.4mm × 255mm × 155 mm; echo time, 2.3 ms; repetition time, 2500 ms; inversion time, 934 ms; flip angle, 7º; GRAPPA acceleration factor, 2. Additionally, this participant underwent a blood draw in 22 sessions. Genomic DNA was isolated from 24 whole blood samples to compute epigenetic clocks in 20 sessions (see Figure 1B and section Epigenetic clocks). MRI data of participant S01 is available in OpenNeuro (https://openneuro.org/datasets/ds005299) and additional information can be found in Pritschet et al. 2024.

Participants S02 (conceived naturally at 34 years old and gave birth vaginally at 39 gestational weeks) and S03 (conceived naturally at 26 years old and gave birth through an emergency c-section at 39 gestational weeks) underwent 14 and 22 MRI sessions, respectively, from pre-conception to one year postpartum. T1-weighted images were obtained at BIC with the following acquisition parameters: voxel size, 0.94mm × 0.94mm × 0.94 mm; field of view, 195.5mm × 240.1mm × 240.1 mm; echo time, 2.22 ms; repetition time, 2500 ms; inversion time, 851 ms; flip angle, 7º; GRAPPA acceleration factor, 2. Additional information can be found in [34].

**Epigenetic clocks**

Genomic DNA was isolated from whole blood samples using a semi-automated magnetic bead approach (Maxwell RSC Blood DNA Kit, Promega) quantified by fluorometry (Quatus, Promega) followed by sodium bisulfite conversion using a commercially available kit (EZ-DNA Methylation Kit, Zymo Research). Genome-wide DNA methylation was described using the Infinium Methylation EPIC array v2 (Illumina) according to the manufacturer's protocol. Quality control and preprocessing of the DNA methylation data were performed using the SeSAMe package. Biological age (PC Clocks), including PCHorvath 1, PCHorvath2, PCPhenoAge, PCGrimAge, and DNAmGrimAge2 were estimated using the methylCIPHER package [35]. The pace of aging (DunedinPACE) estimates were calculated using publicly available code. All analyses were performed in R. For biological age estimates, we compute the biological-age gap as the difference of the estimate and the chronological age.

**Brain age models**

Pretrained brain age models used in this study (*brainageR*, *pyment* and *DeepBrainNet*) were selected from those models validated and publicly-available that showed better accuracy and test-retest reliability in independent studies [14]. Additionally, a recently updated brain age toolbox (brainAGE) was used and trained with publicly available data. For the models yielding brain age prediction, brain age gap (BAG) was computed as the signed difference between the predicted age and the chronological age. Hence, BAG positive values indicate an older age prediction than chronological age, while negative values imply younger age prediction than chronological age. Performance metrics for each brain age model computed on the nulliparous women group of the cohort dataset can be found in Supplementary Table 19.

***BrainageR***: *BrainageR* (https://github.com/james-cole/brainageR) uses a Gaussian Process Regression model to predict age from raw T1-weighted brain MRI images [36]. The image is first segmented and normalized using SPM12. Resulting grey matter, white matter and cerebro-spinal fluid segmentations are then masked and a Principal Component Analysis rotation matrix is applied selecting a total of 435 variables. Gaussian Process Regression is performed with the *kernlab* R package, with a RBF kernel and using default hyperparameters. The model was trained on a curated sample of n = 3377 healthy individuals from publicly-available datasets, with an age range of 18-90 years, a mean age of 40.6 years and a standard deviation of 21.4 years. The specific number of male and female individuals in the training set was not reported. We compute brain age prediction using *brainageR* with a docker container available at https://github.com/fprados/brainageR_dockerfile

***pyment***: *pyment* (https://github.com/estenhl/pyment-public) uses a Simple Fully Convolutional Neural Network with a regression head to predict age from preprocessed T1-weighted brain MRI images[37]. The necessary image preprocessing includes bias field correction and skull-striping using FreeSurfer's *recon-all autorecon-1*, and reorientation of the image array to LAS/RAS. Then, the resulting image is rigidly registered to 1mm MNI152 brain template and, finally, the image is cropped to meet the geometry of the input layer of the neural network. The neural network was trained with a sample of 53542 healthy individuals (27715 female), with ages ranging from 3 to 95 years. We compute brain age estimates with *pyment* using the docker container available at https://hub.docker.com/r/estenhl/sfcn-reg-predict-brain-age.

***DeepBrainNet***: *DeepBrainNet* (https://github.com/vishnubashyam/DeepBrainNet) uses a 2D Convolucional Neural Network to predict age from preprocessed T1-weighted brain MRI images [38]. To predict brain age with DeepBrainNet, we used the implementation in the python package *antspynet* (https://antsx.github.io/ANTsPyNet/docs/build/html/index.html), where image preprocessing with ANTs is already included in the workflow, consisting on n4 bias field correction, brain extraction and affine registration to MNI. The neural network was trained using a sample of n = 11729 healthy participants (6297 female), with ages ranging from 3 to 95 years.

***BrainAGE***: *BrainAGE* (https://github.com/ChristianGaser/BrainAGE) is a toolbox for age prediction using preprocessed T1-weighted brain MRI images, recently updated to use Gaussian Process Regression [39]. In contrast with the above-mentioned tools, brainAGE does not provide a pretrained model, but rather provides the necessary tools to train custom models. In our study, we used images of healthy participants of a set of publicly available dataset, including **OASIS-1** [40], **OASIS-3** [41], **IXI**, **Cam-CAN** [42,43], **SALD** and **eNKI-RS**[44]. When several images of the same participant were available only the first one with good quality was used. T1-weighted images from the training datasets and the maternity cohort and dense-sampling datasets were preprocessed using the default processing of the CAT12.9 Toolbox[45], with affine registration to the MNI152NLin2009cAsym template. Segmented gray matter and white matter volumes (with different levels of resampling and smoothing), along with gender were then used as inputs to train the Gaussian Process Regression model to predict age on the maternity datasets. For training, we selected all participants from the above-mentioned training datasets within an age range from 18 to 60 years, leading to 2268 participants (1354 female), with mean age of 40.02 (SD 13.3) years. For the prediction phase, no age trend correction on BAG was performed using the toolbox.

**Time to parturition:** For each participant, we consider the temporal variable **time-to-parturition** as the time difference between date of parturition and the date when the data was acquired. Hence, positive values of this variable correspond to the postpartum period, while negative values correspond to the pregnancy period or to the pre-conception period. While this variable allows for a temporal matching between participants of their transition to maternity, it might be worth mentioning that the matching is more accurate in the postpartum period than in the pregnancy and pre-conception period due to the different duration of the gestation for each participant. For nulliparous women in the cohort dataset, this time variable is defined by matching randomly chosen values with those of gestational mothers group at the one month postpartum session and then propagating its values in the remaining session using the corresponding intersession time. More specifically, we set values at the above mentioned session by selecting random values drawn from a Gaussian distribution with the same mean and standard deviation as for the gestational mothers. For one nulliparous mother, this matching was done using the mid-pregnancy session, since the one month postpartum session was not available. Additionally, the pre-conception session was acquired several months before the conception occurred for some participants data (in the most extreme case 2.17 years before the conception), leading to time-to-parturition values very far away from the range of interest, and suboptimal placement of the smooth basis in the HGAM analysis. To overcome this, we artificially (exponentially) approached time-to-parturition values lower than -42 weeks to set them in a range between -52 and -42 weeks.

## Statistical Analyses

**Hierarchical Generalized Additive models:** We first fitted HGAM models using the R package *mgcv (version 1.9-1)*[46]*.* to study the temporal dependence of BAG across gestation and postpartum. For the Cohort dataset, we pose the following model:

Brain-age gap ~ s(time-to-parturition, by=group, k=10, bs='ts' )

+ group + age * scanning_site
+ s(participant_id, bs='re')

Here, s(time-to-parturition, by=group, k=10, bs='ts') denotes three independent sets of thin plate spline basis (TPSB), one for each group (gestational mothers, non-gestational mothers and nulliparous women), with 10 basis functions each, allowing for a quite generic fit of the "time-to-parturition" temporal dependence. Additionally, s(participant_id, bs='re'), stands for per-participant random effect smooths, equivalent to random intercepts. We also included "group" as a fixed effect in the model, and mean centered "age", "scanning_site" (Madrid or Barcelona) and its interaction as fixed effect covariates to model the age-bias, which is known to occur in brain age models[47]. Brain age models present an age-bias in the estimated BAG, which makes necessary the inclusion of age as a covariate in the models. Although in some cases this may limit the interpretation of the bias-corrected metrics[48], our longitudinal design overcomes this limitation by tracking brain age changes within the same individuals, reducing the confounding effects of cross-sectional age differences.

For the dense-sampling dataset, the model was:

Brain-age gap ~ s(time-to-parturition, k=10, bs='ts' )
+ scanning_site + s(participant_id, bs='re').

Here, we only have one set of TPSB, with 10 functions, for the "time-to-parturition" temporal dependence. "scanning_site" was BIC or FIBRE. Note that for this dataset we did not include "age" as covariate since only three participants are available and there is no control group.

These models were used independently for the "brain-age gap" computed from the four brain-age models, *BrainageR*, *pyment*, *DeepBrainNet*, and *brainAGE*. In all models we assume a Gaussian distribution of errors and were fitted using restricted maximum likelihood. For each fitted model, we tested for the overall significance of the temporal smooths using ANOVA tests. For each brain-age model and dataset obtained p-values were False Discovery Rate (FDR) corrected independently using the Benjamini-Hochberg method [49].

**Linear Mixed Effects Models:** Next, we fitted LMEM models using again the *mgcv* R package to allow for brain-age gap comparison between groups and sessions. For the Cohort dataset we pose the model:

Brain-age gap ~ group * session + age * scanning_site
+ s(participant_id, bs='re').

Here, we include "group", "session" and its interaction as fixed effects of interest. "session" is a categorical variable coding to which of the six sessions corresponds to each observation. As in the HGAM models, we include per-participant random smooths (equivalent to random intercepts) and "age", "scanning_site" and its interaction as covariates. To test the effect of parturition type, we apply the same model but with "group" defined as the type of parturition with categories: vaginal parturition, emergency c-section, scheduled c-section and control, which includes nulliparous women and non-gestational mothers. For the dense-sampling dataset we first grouped the observations in seven gestational/postpartum periods: (1) pre-conception; (2) 1st gestational trimester; (3) 2nd gestational trimester; (4) 3rd gestational trimester; (5) early postpartum: up to 8 weeks postpartum; (6) ~6 months postpartum: from 8 weeks to 30 weeks postpartum; (7) ~1 year postpartum: more than 30 weeks postpartum. Then, we used the following model:

$$\text{Brain-age gap} \sim \text{gestation/postpartum period} + \text{scanning\_site} + s(\text{participant\_id}, bs='re')$$

As for HGAM models, these LMEM models were used independently for the "brain-age gap" computed from the four brain-age models assuming Gaussian distributed errors. Once the models were fitted, we used the *emmeans* R package to perform comparisons. For the cohort dataset, independently for each group, we tested for differences between the pre-conception session (ses-1) and the other five following sessions, and between the late pregnancy session (ses-3) and the three postpartum sessions, yielding a total of 24 comparisons/tests per brain-age model. For birth type comparisons, we conducted 116 statistical tests comprising within-group and between-group comparisons across both longitudinal and cross-sectional analyses. For the dense-sampling dataset, we tested for differences between the pre-conception period with the six following periods, and between the 3rd gestational trimester period and the three postpartum periods, yielding a total of 9 comparisons per brain-age model. For each brain-age model and dataset obtained p-values were FDR corrected independently using the Benjamini-Hochberg method[49].

**Brain-age and biological clocks correlations:**

Focusing on the data of participant S01, we computed pair-wise Pearson correlation of the twelve metrics, that include four brain-age gap metrics, five biological-age gap metrics, and one pace of aging metric. All the brain-age gap metrics were defined in 27 temporal points, but for *brainageR* that was defined in 26. Biological-age gap metrics and pace of aging were defined in 20 temporal points. The average was computed for the four sessions in which we had two measurements of these metrics. The degrees of freedom of each pair-wise correlation are reported in Supplementary Table 18. Obtained p-values were FDR corrected using the Benjamini-Hochberg method[49]. Additionally, GAMs were fitted for each metric separately (brain-age gap and biological-age gap) including only a time-to-parturition TPSB temporal smooth.

## ACKNOWLEDGEMENTS

We thank all the women who participated in the study. We are particularly grateful to the first-time mothers for sharing with us such a special period as the birth of their first child.
The project leading to these results has received funding from the Instituto de Salud Carlos III (project PI22/01365), Ministerio de Ciencia, Innovación y Universidades (project RTI2018-093952-B-100), 'la Caixa' Foundation under project code LCF/PR/HR19/52160001, the Chan Zuckerberg Initiative (grant number CZIF2024-010398), the European Research Council under the 'European Union's Horizon 2020' research and innovation programme (grant agreement no. 883069), and was co-funded by the European Regional Development Fund (ERDF), 'A way of making Europe.' M.M.G is supported by The Ann S. Bowers Women's Brain Health Initiative, an International Postdoctoral Fellowship from the Ramon Areces Foundation, an International

Training Grant from the Society for Reproductive Investigation, and a NARSAD Young Investigator Grant from the Brain & Behavior Research Foundation.
We acknowledge the usage of the following dataset for training brainAGE:
OASIS-1: OASIS-1: Cross-Sectional: Principal Investigators: D. Marcus, R, Buckner, J, Csernansky J. Morris; P50 AG05681, P01 AG03991, P01 AG026276, R01 AG021910, P20 MH071616, U24 RR021382.
OASIS-3: OASIS-3: Longitudinal Multimodal Neuroimaging: Principal Investigators: T. Benzinger, D. Marcus, J. Morris; NIH P30 AG066444, P50 AG00561, P30 NS09857781, P01 AG026276, P01 AG003991, R01 AG043434, UL1 TR000448, R01 EB009352. AV-45 doses were provided by Avid Radiopharmaceuticals, a wholly owned subsidiary of Eli Lilly.
CamCAN: Data collection and sharing for this project was provided by the Cambridge Centre for Ageing and Neuroscience (CamCAN). CamCAN funding was provided by the UK Biotechnology and Biological Sciences Research Council (grant number BB/H008217/1), together with support from the UK Medical Research Council and University of Cambridge, UK.

## DATA AVAILABILITY

IXI dataset : This data is made available under the Creative Commons CC BY-SA 3.0 license. If you use the IXI data please acknowledge the source of the IXI data, e.g. this website (https://brain-development.org/ixi-dataset/).
Cam-CAN dataset: Data used in the preparation of this work were obtained from the CamCAN repository (available at http://www.mrc-cbu.cam.ac.uk/datasets/camcan/), (Taylor et al., 2016, Shafto et al., 2015)
SALD dataset: Data available at https://fcon_1000.projects.nitrc.org/indi/retro/sald.html
eNKI dataset: Data available at https://fcon_1000.projects.nitrc.org/indi/pro/eNKI_RS_TRT/FrontPage.html or https://rocklandsample.org/accessing-the-neuroimaging-data-releases
OASIS-1 and OASIS-3 datasets: Access to these datasets can be requested at https://sites.wustl.edu/oasisbrains/datasets/

## SUPPLEMENTARY MATERIAL

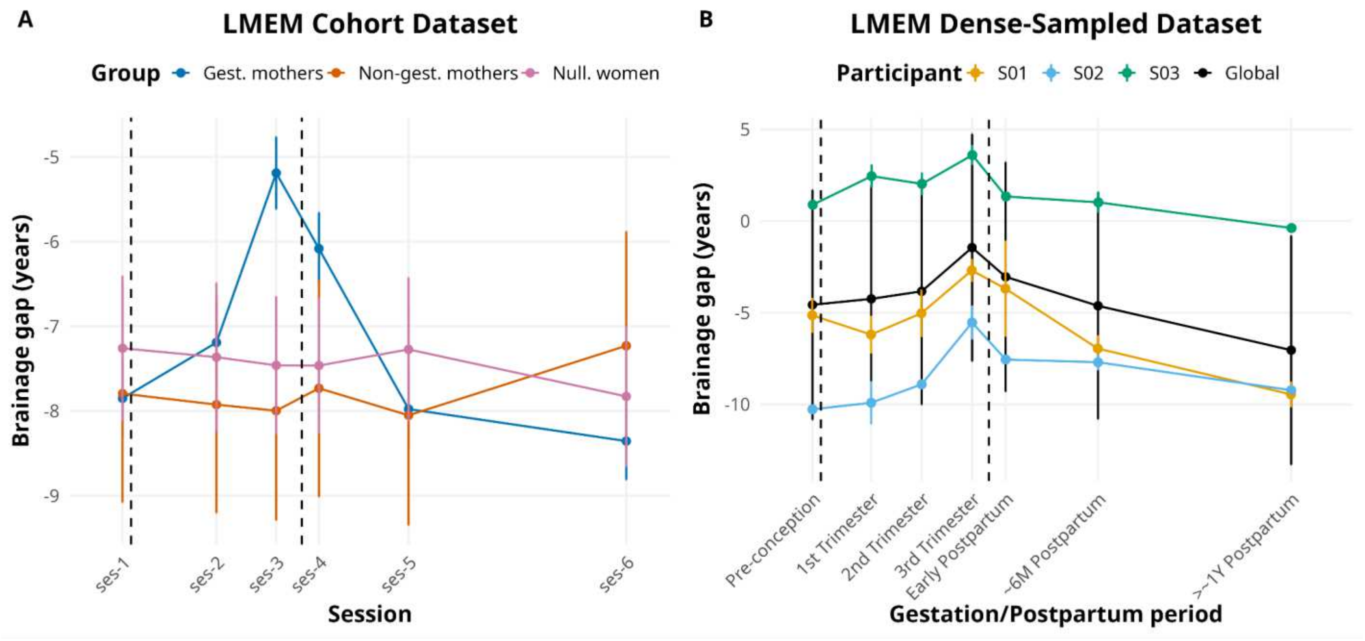

**Supplementary Figure 1.** Brain age gap trajectories obtained using Linear Mixed effects models for *BrainageR*. A) Cohort dataset; B) Dense-sampled dataset with binned data in seven gestation/postpartum periods. Dashed vertical lines indicate nominal times for conception (x=-40) and parturition (x=0). Error bars represent 95% confidence intervals for the estimated marginal means.

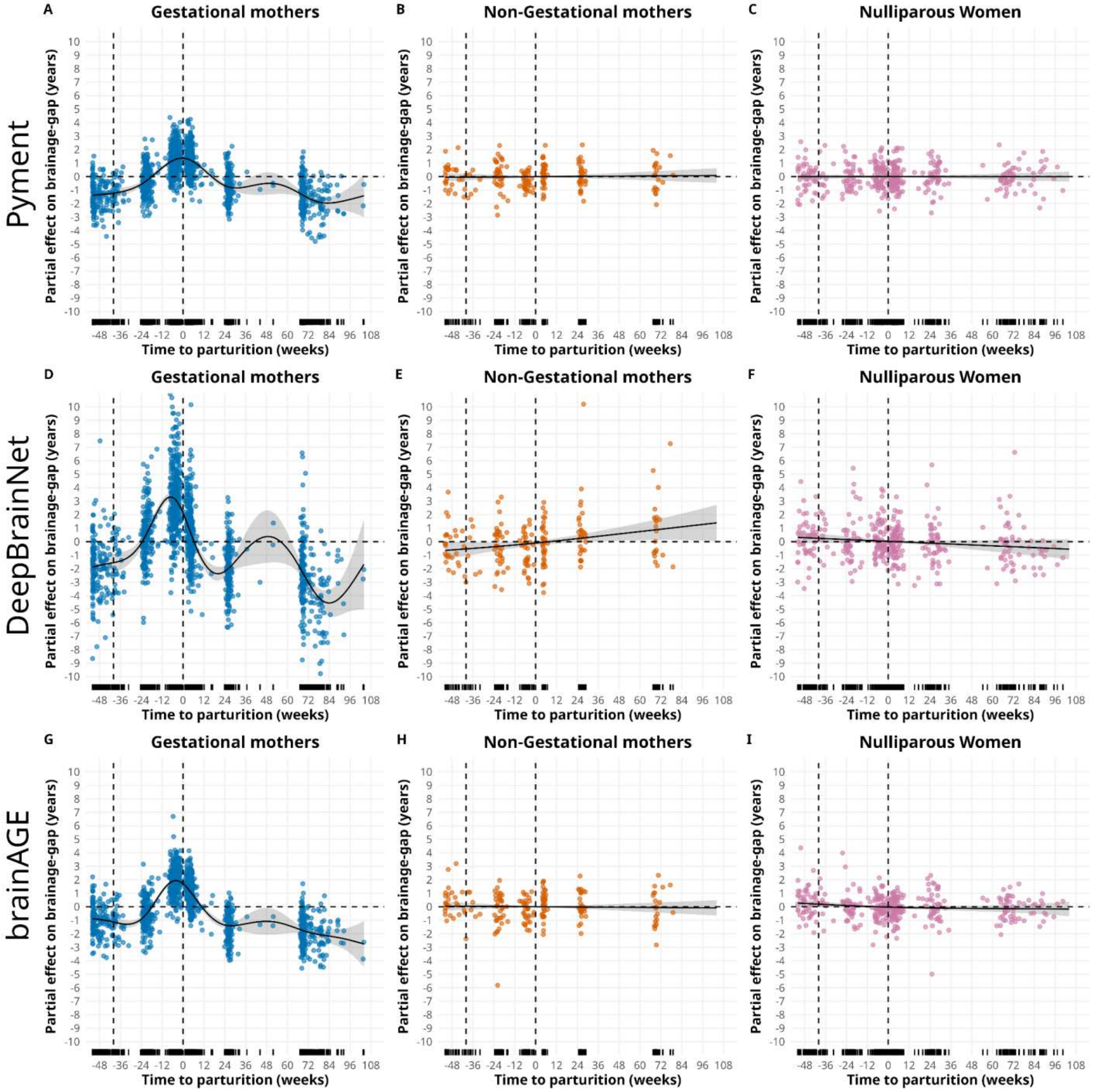

**Supplementary Figure 2.** Brain aging trajectories in the cohort dataset estimated using Hierarchical Generalized Additive Models (HGAM) with the *Pyment*, *DeepBrainNet*, and *BrainAge* algorithms. Dashed vertical lines indicate nominal times for conception (x=-40) and parturition (x=0). Mean temporal smooth trajectories are shown in black and gray shadows indicating pointwise 95% confidence intervals.

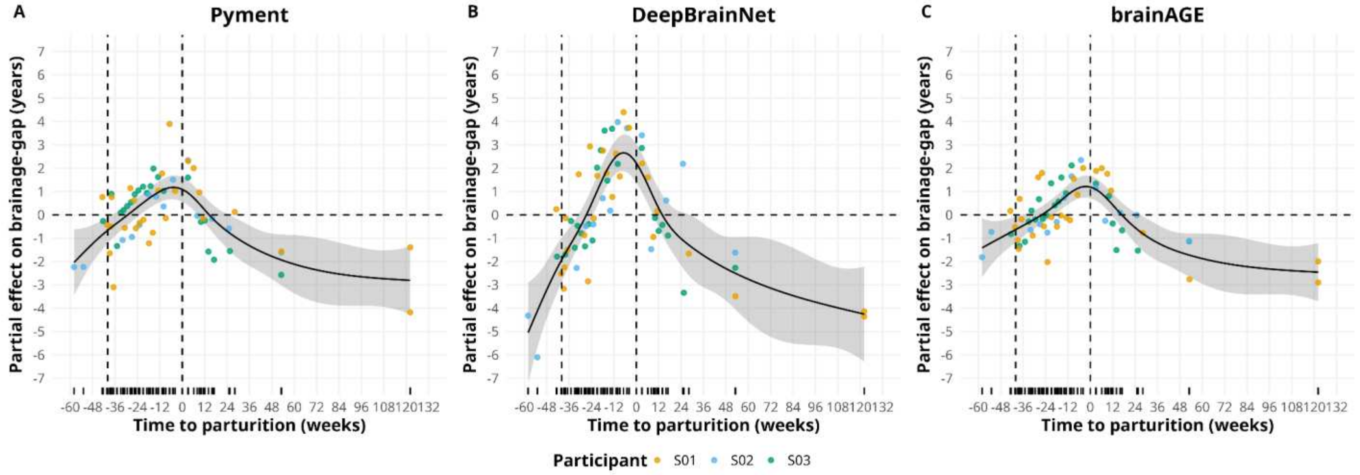

**Supplementary Figure 3.** Brain aging trajectories in the dense-sampling dataset estimated using Hierarchical Generalized Additive Models (HGAM) with the *Pyment*, *DeepBrainNet*, and *BrainAge* algorithms. Dashed vertical lines indicate nominal times for conception (x=-40) and parturition (x=0). Mean temporal smooth trajectories are shown in black and gray shadows indicating pointwise 95% confidence intervals. Each color represents a different subject.

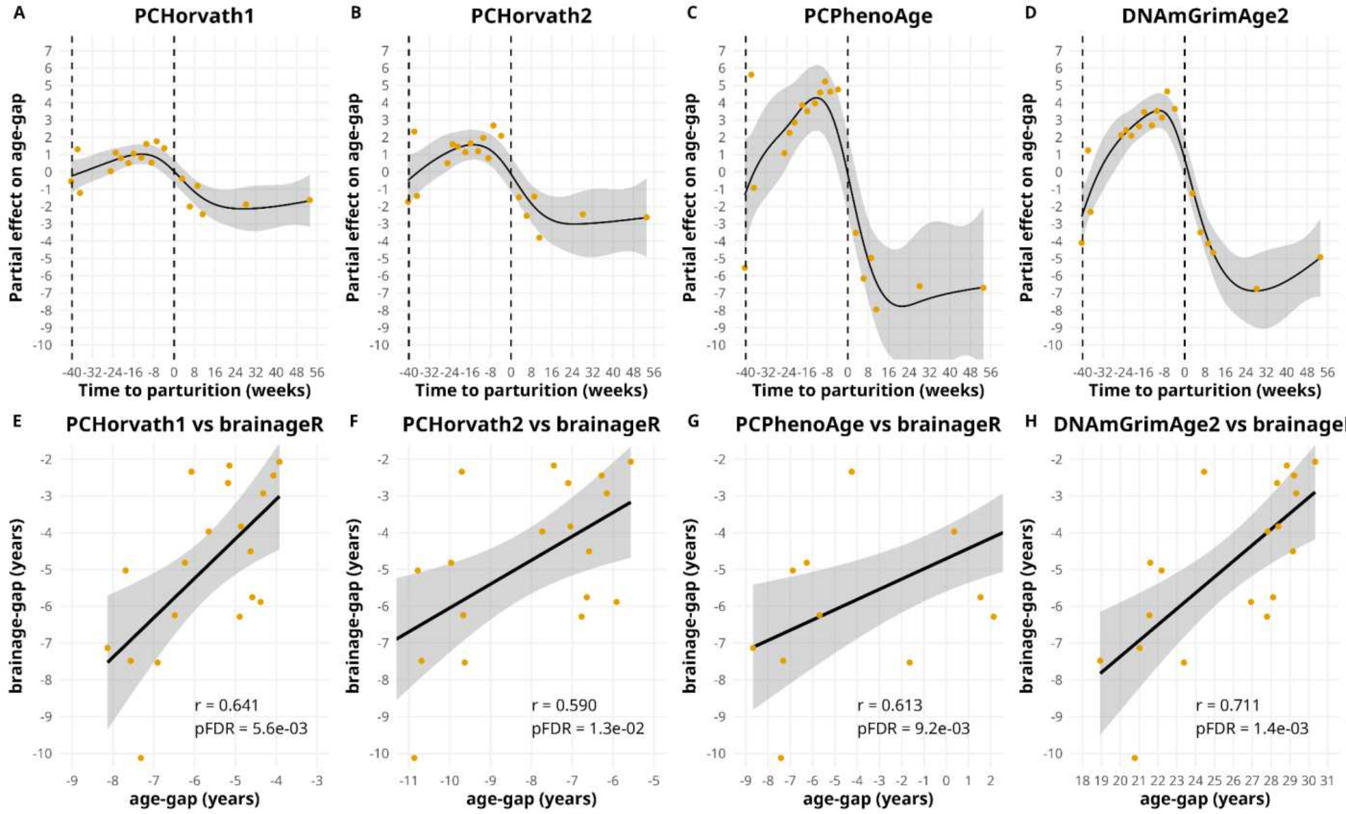

**Supplementary Figure 4.** Trajectories of additional epigenetic clocks in the dense-sampled participant estimated using Hierarchical Generalized Additive Models (HGAM). Gray shadows represent the 95% confidence interval, r is the Pearson correlation coefficient" and pFDR is the associated family-wise rate corrected p-value.

**Supplementary Table 1.** Participant distribution and session-level variables.

**Barcelona cohort**

| **Characteristic** | **N** | ses-1[1] | ses-2[1] | ses-3[1] | ses-4[1] | ses-5[1] | ses-6[1] |
|---|---|---|---|---|---|---|---|
| Group | 1,571 | | | | | | |
| Gestational Mothers | | 180 (66%) | 182 (70%) | 206 (74%) | 212 (70%) | 171 (68%) | 148 (71%) |
| Non-Gestational Mothers | | 35 (13%) | 36 (14%) | 31 (11%) | 35 (12%) | 30 (12%) | 21 (10%) |
| Nulliparous Women | | 57 (21%) | 42 (16%) | 40 (14%) | 57 (19%) | 50 (20%) | 38 (18%) |
| Gestational weeks | 450 | | 18.5 (1.3) | 34.4 (1.1) | | | |
| Gestational weeks [Gestational Mothers] | 384 | | 18.6 (1.3) | 34.4 (1.1) | | | |
| Gestational weeks [Non-Gestational Mothers] | 66 | | 18.4 (0.8) | 34.4 (1.0) | | | |
| Time from parturition [weeks] | 1,287 | -61.2 (24.3) | -20.8 (2.3) | -5.2 (1.7) | 5.0 (1.7) | 26.5 (1.9) | 69.4 (3.8) |
| Time from parturition [weeks] [Gestational Mothers] | 1,099 | -62.1 (25.0) | -20.9 (2.2) | -5.3 (1.7) | 5.0 (1.8) | 26.5 (2.0) | 69.2 (3.9) |
| Time from parturition [weeks] [Non-Gestational Mothers] | 188 | -56.7 (20.5) | -20.6 (2.6) | -5.1 (1.7) | 4.7 (0.6) | 26.4 (1.1) | 70.2 (2.9) |

[1]n (%); Mean (SD)

**Madrid Cohort**

| **Characteristic** | **N** | ses-3[1] | ses-4[1] | ses-6[1] |
|---|---|---|---|---|
| Group | 372 | | | |
| Gestational Mothers | | 113 (77%) | 112 (78%) | 64 (77%) |
| Nulliparous Women | | 33 (23%) | 31 (22%) | 19 (23%) |
| Gestational weeks [Gestational Mothers] | 113 | 36.2 (0.9) | | |
| Time from parturition [weeks] [Gestational Mothers] | 289 | -3.7 (1.3) | 3.1 (1.4) | 79.8 (5.9) |

[1]n (%); Mean (SD)

**Supplementary Table 2.** General demographic characteristics of the full cohort dataset.

| **Characteristic** | Gestational Mother[1] | Non-Gestational Mother[1] | Nulliparous Women[1] | **p-value**[2] |
|---|---|---|---|---|
| Acquisition Site [nº of participants (%)] | | | | <0.001 |
| Barcelona | 226 (66%) | 37 (100%) | 58 (64%) | |
| Madrid | 114 (34%) | 0 (0%) | 33 (36%) | |

| | | | | |
|---|---|---|---|---|
| Age at ses-4 [years] | 34.4 (4.2) | 33.8 (3.8) | 32.9 (4.1) | 0.005 |
| Unknown | 6 | 2 | 1 | |
| Estimated IQ at ses-4 (WAIS-IV Digits Span) | 28.5 (5.3) | 27.7 (5.8) | 29.8 (4.4) | 0.11 |
| Unknown | 9 | 2 | 4 | |
| Education [nº of participants (%)] | | | | 0.2 |
| none | 0 (0%) | 1 (2.8%) | 0 (0%) | |
| primary | 9 (2.6%) | 0 (0%) | 0 (0%) | |
| secondary | 33 (9.7%) | 3 (8.3%) | 9 (9.9%) | |
| higher | 298 (88%) | 32 (89%) | 82 (90%) | |
| Unknown | 0 | 1 | 0 | |
| Monthly Income [euros/month] [nº of participants (%)] | | | | <0.001 |
| <600 | 3 (0.9%) | 0 (0%) | 2 (2.4%) | |
| 601-1200 | 8 (2.4%) | 0 (0%) | 9 (11%) | |
| 1201-2000 | 59 (18%) | 2 (5.6%) | 28 (34%) | |
| 2001-3000 | 96 (28%) | 11 (31%) | 23 (28%) | |
| 3001-4500 | 127 (38%) | 17 (47%) | 14 (17%) | |
| >4500 | 44 (13%) | 6 (17%) | 7 (8.4%) | |
| Unknown | 3 | 1 | 8 | |

[1]n (%); Mean (SD)

[2]Pearson's Chi-squared test; Kruskal-Wallis rank sum test; Fisher's exact test

**Supplementary Table 3.** Perinatal demographics of gestational mothers in the cohort dataset.

| **Characteristic** | Gestational Mother[1] |
|---|---|
| Assisted reproduction [nº of participants (%)] | |
| Natural | 220 (65%) |
| Assisted | 119 (35%) |
| Unknown | 1 |
| Assisted reproduction method [nº of participants (%)] | |
| AI | 25 (7.4%) |
| ICSI | 46 (14%) |
| IVF | 48 (14%) |
| natural | 220 (65%) |
| Unknown | 1 |
| Gestational weeks at delivery [weeks] | 39.72 (1.46) |
| Unknown | 4 |
| Type of parturition [nº of participants (%)] | |
| c-section emergency | 61 (19%) |
| c-section scheduled | 30 (9.2%) |
| vaginal | 236 (72%) |
| Unknown | 13 |
| Type of Baby Feeding at ses-4 [nº of participants (%)] | |
| breast | 264 (80%) |
| formula | 22 (6.7%) |
| mixed | 42 (13%) |
| Unknown | 12 |

[1]n (%); Mean (SD)

**Supplementary Table 4.** HGAM coefficients and smooth terms for the cohort dataset using *brainageR*.

| Coefficient | Estimate | Std.Error | t.value | p.value |
|---|---|---|---|---|

| | | | | |
|---|---|---|---|---|
| (Intercept) | -7.35 | 0.32 | -22.93 | <0.001 |
| Group [Non-Gestational Mother] | -0.95 | 0.63 | -1.51 | 0.131 |
| Group [Nulliparous Women] | -0.54 | 0.43 | -1.26 | 0.209 |
| Age | -0.60 | 0.07 | -8.96 | <0.001 |
| Dataset [Barcelona] | 0.833 | 0.374 | 0.374 | 0.026 |
| Age : Dataset [Barcelona] | 0.04 | 0.08 | 0.50 | 0.618 |

| Smooth Term | edf | Ref.df | F | p.value | p.FDR |
|---|---|---|---|---|---|
| s(Time to Parturition) : Group [Gestational Mother] | 8.07 | 8.68 | 97.84 | <0.001 | <0.001 |
| s(Time to Parturition) : Group [Non-Gestational Mother] | 1.94 | 2.37 | 1.27 | 0.241 | 0.241 |
| s(Time to Parturition) : Group [Nulliparous Women] | 1.00 | 1.00 | 2.87 | 0.090 | 0.135 |
| s(Participant) | 442.98 | 464.00 | 25.60 | <0.001 | - |

Deviance explained = 0.927, $R^2$(Adjusted) = 0.904, n = 1943

**Supplementary Table 5.** LMEM brain age gap estimates of the cohort dataset derived from *BrainageR*. Include statistics comparing each timepoint to baseline, as well as change from late pregnancy to 6 months postpartum. Include three groups.

| Coefficient | Estimate | Std.Error | t.value | p.value |
|---|---|---|---|---|
| (Intercept) | -8.182 | 0.343 | -23.889 | <0.001 |
| Group [Non-Gestational Mother] | 0.056 | 0.673 | 0.083 | 0.933 |
| Group [Nulliparous Women] | 0.592 | 0.477 | 1.241 | 0.215 |
| Session [ses-2] | 0.659 | 0.152 | 4.332 | <0.001 |
| Session [ses-3] | 2.661 | 0.149 | 17.898 | <0.001 |
| Session [ses-4] | 1.768 | 0.150 | 11.801 | <0.001 |
| Session [ses-5] | -0.127 | 0.171 | -0.740 | 0.459 |
| Session [ses-6] | -0.505 | 0.184 | -2.738 | 0.006 |
| Age | -0.565 | 0.064 | -8.843 | <0.001 |
| Dataset [Barcelona] | 0.663 | 0.371 | 1.786 | 0.074 |
| Group [Non-Gestational Mother] : Session [ses-2] | -0.790 | 0.363 | -2.174 | 0.030 |
| Group [Nulliparous Women] : Session [ses-2] | -0.765 | 0.325 | -2.350 | 0.019 |
| Group [Non-Gestational Mother] : Session [ses-3] | -2.863 | 0.375 | -7.642 | <0.001 |
| Group [Nulliparous Women] : Session [ses-3] | -2.863 | 0.299 | -9.563 | <0.001 |
| Group [Non-Gestational Mother] : Session [ses-4] | -1.706 | 0.362 | -4.707 | <0.001 |
| Group [Nulliparous Women] : Session [ses-4] | -1.973 | 0.283 | -6.978 | <0.001 |
| Group [Non-Gestational Mother] : Session [ses-5] | -0.130 | 0.384 | -0.338 | 0.735 |
| Group [Nulliparous Women] : Session [ses-5] | 0.113 | 0.313 | 0.360 | 0.719 |
| Group [Non-Gestational Mother] : Session [ses-6] | 1.070 | 0.426 | 2.511 | 0.012 |
| Group [Nulliparous Women] : Session [ses-6] | -0.064 | 0.315 | -0.204 | 0.838 |
| Age : Dataset [Barcelona] | -0.013 | 0.074 | -0.175 | 0.861 |

| Smooth | edf | Ref.df | F | p.value |
|---|---|---|---|---|
| s(Participant ID) | 442.98 | 464.00 | 25.469 | <0.001 |

Deviance explained = 0.927, $R^2$(Adjusted) = 0.904, n = 1943

| Group | Contrast | Estimate | SE | df | t.ratio | p.value | pFDR |
|---|---|---|---|---|---|---|---|
| Gestational Mother | (ses-2) - (ses-1) | 0.659 | 0.152 | 1479.02 | 4.332 | <0.001 | <0.001 |
| Gestational Mother | (ses-3) - (ses-1) | 2.661 | 0.149 | 1479.02 | 17.898 | <0.001 | <0.001 |

| | | | | | | | |
|---|---|---|---|---|---|---|---|
| Gestational Mother | (ses-4) - (ses-1) | 1.768 | 0.150 | 1479.02 | 11.801 | <0.001 | <0.001 |
| Gestational Mother | (ses-5) - (ses-1) | -0.127 | 0.171 | 1479.02 | -0.740 | 0.459 | 0.699 |
| Gestational Mother | (ses-6) - (ses-1) | -0.505 | 0.184 | 1479.02 | -2.738 | 0.006 | 0.021 |
| Gestational Mother | (ses-4) - (ses-3) | -0.893 | 0.111 | 1479.02 | -8.063 | <0.001 | <0.001 |
| Gestational Mother | (ses-5) - (ses-3) | -2.788 | 0.142 | 1479.02 | -19.683 | <0.001 | <0.001 |
| Gestational Mother | (ses-6) - (ses-3) | -3.166 | 0.141 | 1479.02 | -22.466 | <0.001 | <0.001 |
| Non-Gestational Mother | (ses-2) - (ses-1) | -0.131 | 0.333 | 1479.02 | -0.392 | 0.695 | 0.861 |
| Non-Gestational Mother | (ses-3) - (ses-1) | -0.202 | 0.351 | 1479.02 | -0.576 | 0.564 | 0.753 |
| Non-Gestational Mother | (ses-4) - (ses-1) | 0.063 | 0.340 | 1479.02 | 0.185 | 0.853 | 0.962 |
| Non-Gestational Mother | (ses-5) - (ses-1) | -0.257 | 0.360 | 1479.02 | -0.713 | 0.476 | 0.699 |
| Non-Gestational Mother | (ses-6) - (ses-1) | 0.565 | 0.413 | 1479.02 | 1.366 | 0.172 | 0.376 |
| Non-Gestational Mother | (ses-4) - (ses-3) | 0.265 | 0.347 | 1479.02 | 0.764 | 0.445 | 0.699 |
| Non-Gestational Mother | (ses-5) - (ses-3) | -0.054 | 0.365 | 1479.02 | -0.149 | 0.881 | 0.962 |
| Non-Gestational Mother | (ses-6) - (ses-3) | 0.767 | 0.415 | 1479.02 | 1.846 | 0.065 | 0.173 |
| Nulliparous Women | (ses-2) - (ses-1) | -0.105 | 0.291 | 1479.02 | -0.362 | 0.717 | 0.861 |
| Nulliparous Women | (ses-3) - (ses-1) | -0.202 | 0.271 | 1479.02 | -0.746 | 0.456 | 0.699 |
| Nulliparous Women | (ses-4) - (ses-1) | -0.205 | 0.253 | 1479.02 | -0.808 | 0.419 | 0.699 |
| Nulliparous Women | (ses-5) - (ses-1) | -0.014 | 0.282 | 1479.02 | -0.049 | 0.961 | 0.991 |
| Nulliparous Women | (ses-6) - (ses-1) | -0.569 | 0.294 | 1479.02 | -1.934 | 0.053 | 0.160 |
| Nulliparous Women | (ses-4) - (ses-3) | -0.003 | 0.225 | 1479.02 | -0.012 | 0.991 | 0.991 |
| Nulliparous Women | (ses-5) - (ses-3) | 0.188 | 0.276 | 1479.02 | 0.682 | 0.495 | 0.699 |
| Nulliparous Women | (ses-6) - (ses-3) | -0.367 | 0.264 | 1479.02 | -1.390 | 0.165 | 0.376 |

**Supplementary Table 6.** HGAM coefficients and smooth terms for the dense-sampling dataset using *brainageR.*

| Coefficient | Estimate | Std.Error | t.value | p.value |
|---|---|---|---|---|
| (Intercept) | -4.07 | 3.04 | -1.34 | 0.187 |
| Scanning Site [UCSB] | 0.14 | 0.69 | 0.20 | 0.844 |

| Smooth Term | edf | Ref.df | F | p.value | p.FDR |
|---|---|---|---|---|---|
| s(Time to Parturition) | 5.40 | 6.37 | 9.09 | <0.001 | <0.001 |
| s(Participant) | 1.99 | 2.00 | 254.98 | <0.001 | - |

Deviance explained = 0.932, $R^2$(Adjusted) = 0.922, n = 62

**Supplementary Table 7.** LMEM brain age gap estimates of the dense-sampling dataset derived from *BrainageR*.

| Coefficient | Estimate | Std.Error | t.value | p.value |
|---|---|---|---|---|
| (Intercept) | -4.629 | 3.184 | -1.454 | 0.152 |
| Gestation/Postpartum [1st Trimester] | 0.325 | 0.622 | 0.522 | 0.604 |
| Gestation/Postpartum [2nd Trimester] | 0.736 | 0.661 | 1.112 | 0.271 |
| Gestation/Postpartum [3rd Trimester] | 3.122 | 0.705 | 4.427 | <0.001 |
| Gestation/Postpartum [Early Postpartum] | 1.525 | 0.829 | 1.840 | 0.072 |
| Gestation/Postpartum [~6 Months Postpartum] | -0.055 | 0.671 | -0.082 | 0.935 |
| Gestation/Postpartum [>1 Year Postpartum] | -2.474 | 0.740 | -3.344 | 0.002 |
| Scanning Site [UCSB] | 0.125 | 0.649 | 0.192 | 0.848 |

| Smooth | edf | Ref.df | F | p.value |
|---|---|---|---|---|

| | | | | |
|---|---|---|---|---|
| s(Participant ID) | 1.99 | 2.00 | 301.132 | <0.001 |

Deviance explained = 0.940, R²(Adjusted) = 0.930, n = 62

| Contrast | Estimate | SE | df | t.ratio | p.value | pFDR |
|---|---|---|---|---|---|---|
| 1st Trimester - Pre-conception | 0.325 | 0.622 | 52.01 | 0.522 | 0.604 | 0.679 |
| 2nd Trimester - Pre-conception | 0.736 | 0.661 | 52.01 | 1.112 | 0.271 | 0.349 |
| 3rd Trimester - Pre-conception | 3.122 | 0.705 | 52.01 | 4.427 | <0.001 | <0.001 |
| Early Postpartum - Pre-conception | 1.525 | 0.829 | 52.01 | 1.840 | 0.072 | 0.107 |
| 6 Months Postpartum - Pre-conception | -0.055 | 0.671 | 52.01 | -0.082 | 0.935 | 0.935 |
| 1 Year Postpartum - Pre-conception | -2.474 | 0.740 | 52.01 | -3.344 | 0.002 | 0.003 |
| Early Postpartum - 3rd Trimester | -1.596 | 0.715 | 52.01 | -2.232 | 0.030 | 0.054 |
| 6 Months Postpartum - 3rd Trimester | -3.177 | 0.544 | 52.01 | -5.838 | <0.001 | <0.001 |
| 1 Year Postpartum - 3rd Trimester | -5.596 | 0.675 | 52.01 | -8.292 | <0.001 | <0.001 |

**Supplementary Table 8.** LMEM brain age gap estimates of the cohort dataset derived from *BrainageR* including as covariates conception method and mode of delivery.

| Coefficient | Estimate | Std.Error | t.value | p.value |
|---|---|---|---|---|
| (Intercept) | -7.980 | 0.366 | -21.784 | <0.001 |
| Birth Type [Emergency C-Section] | -0.254 | 0.582 | -0.436 | 0.663 |
| Birth Type [Scheduled C-Section] | -0.926 | 0.786 | -1.179 | 0.239 |
| Birth Type [Control] | 0.250 | 0.442 | 0.566 | 0.571 |
| Session [ses-2] | 0.564 | 0.192 | 2.943 | 0.003 |
| Session [ses-3] | 2.447 | 0.182 | 13.431 | <0.001 |
| Session [ses-4] | 1.611 | 0.183 | 8.827 | <0.001 |
| Session [ses-5] | -0.199 | 0.210 | -0.949 | 0.343 |
| Session [ses-6] | -0.735 | 0.219 | -3.357 | <0.001 |
| Age | -0.578 | 0.064 | -9.040 | <0.001 |
| Dataset [Barcelona] | 0.624 | 0.370 | 1.687 | 0.092 |
| Birth Type [Emergency C-Section] : Session [ses-2] | 0.188 | 0.364 | 0.516 | 0.606 |
| Birth Type [Scheduled C-Section] : Session [ses-2] | 0.505 | 0.516 | 0.979 | 0.328 |
| Birth Type [Control] : Session [ses-2] | -0.702 | 0.288 | -2.439 | 0.015 |
| Birth Type [Emergency C-Section] : Session [ses-3] | 0.352 | 0.343 | 1.025 | 0.305 |
| Birth Type [Scheduled C-Section] : Session [ses-3] | 0.821 | 0.485 | 1.693 | 0.091 |
| Birth Type [Control] : Session [ses-3] | -2.627 | 0.273 | -9.640 | <0.001 |
| Birth Type [Emergency C-Section] : Session [ses-4] | 0.128 | 0.338 | 0.379 | 0.705 |
| Birth Type [Scheduled C-Section] : Session [ses-4] | 0.625 | 0.480 | 1.302 | 0.193 |
| Birth Type [Control] : Session [ses-4] | -1.724 | 0.262 | -6.578 | <0.001 |
| Birth Type [Emergency C-Section] : Session [ses-5] | -0.161 | 0.375 | -0.430 | 0.667 |
| Birth Type [Scheduled C-Section] : Session [ses-5] | 0.936 | 0.522 | 1.794 | 0.073 |
| Birth Type [Control] : Session [ses-5] | 0.074 | 0.291 | 0.256 | 0.798 |
| Birth Type [Emergency C-Section] : Session [ses-6] | 0.392 | 0.363 | 1.080 | 0.280 |
| Birth Type [Scheduled C-Section] : Session [ses-6] | 1.147 | 0.508 | 2.258 | 0.024 |
| Birth Type [Control] : Session [ses-6] | 0.487 | 0.295 | 1.649 | 0.099 |
| Age : Dataset [Barcelona] | 0.011 | 0.074 | 0.144 | 0.885 |

| Smooth | edf | Ref.df | F | p.value |
|---|---|---|---|---|
| s(Participant ID) | 428.47 | 449.00 | 25.232 | <0.001 |

Deviance explained = 0.926, R²(Adjusted) = 0.902, n = 1886

| Birth Type | Contrast | Estimate | SE | df | t.ratio | p.value | pFDR |
|---|---|---|---|---|---|---|---|
| Vaginal Birth | (ses-2) - (ses-1) | 0.564 | 0.192 | 1430.53 | 2.943 | 0.003 | 0.007 |
| Vaginal Birth | (ses-3) - (ses-1) | 2.447 | 0.182 | 1430.53 | 13.431 | <0.001 | <0.001 |
| Vaginal Birth | (ses-4) - (ses-1) | 1.611 | 0.183 | 1430.53 | 8.827 | <0.001 | <0.001 |
| Vaginal Birth | (ses-5) - (ses-1) | -0.199 | 0.210 | 1430.53 | -0.949 | 0.343 | 0.457 |
| Vaginal Birth | (ses-6) - (ses-1) | -0.735 | 0.219 | 1430.53 | -3.357 | <0.001 | 0.002 |
| Vaginal Birth | (ses-4) - (ses-3) | -0.837 | 0.132 | 1430.53 | -6.331 | <0.001 | <0.001 |
| Vaginal Birth | (ses-5) - (ses-3) | -2.647 | 0.176 | 1430.53 | -15.070 | <0.001 | <0.001 |
| Vaginal Birth | (ses-6) - (ses-3) | -3.183 | 0.168 | 1430.53 | -18.937 | <0.001 | <0.001 |
| Emergency C-Section | (ses-2) - (ses-1) | 0.752 | 0.314 | 1430.53 | 2.391 | 0.017 | 0.030 |
| Emergency C-Section | (ses-3) - (ses-1) | 2.799 | 0.302 | 1430.53 | 9.268 | <0.001 | <0.001 |
| Emergency C-Section | (ses-4) - (ses-1) | 1.739 | 0.299 | 1430.53 | 5.815 | <0.001 | <0.001 |
| Emergency C-Section | (ses-5) - (ses-1) | -0.361 | 0.331 | 1430.53 | -1.090 | 0.276 | 0.420 |
| Emergency C-Section | (ses-6) - (ses-1) | -0.343 | 0.330 | 1430.53 | -1.039 | 0.299 | 0.435 |
| Emergency C-Section | (ses-4) - (ses-3) | -1.060 | 0.262 | 1430.53 | -4.047 | <0.001 | <0.001 |
| Emergency C-Section | (ses-5) - (ses-3) | -3.160 | 0.305 | 1430.53 | -10.358 | <0.001 | <0.001 |
| Emergency C-Section | (ses-6) - (ses-3) | -3.142 | 0.289 | 1430.53 | -10.891 | <0.001 | <0.001 |
| Scheduled C-Section | (ses-2) - (ses-1) | 1.069 | 0.481 | 1430.53 | 2.222 | 0.026 | 0.045 |
| Scheduled C-Section | (ses-3) - (ses-1) | 3.268 | 0.455 | 1430.53 | 7.176 | <0.001 | <0.001 |
| Scheduled C-Section | (ses-4) - (ses-1) | 2.236 | 0.451 | 1430.53 | 4.955 | <0.001 | <0.001 |
| Scheduled C-Section | (ses-5) - (ses-1) | 0.736 | 0.489 | 1430.53 | 1.507 | 0.132 | 0.211 |
| Scheduled C-Section | (ses-6) - (ses-1) | 0.412 | 0.482 | 1430.53 | 0.856 | 0.392 | 0.502 |
| Scheduled C-Section | (ses-4) - (ses-3) | -1.033 | 0.370 | 1430.53 | -2.794 | 0.005 | 0.010 |
| Scheduled C-Section | (ses-5) - (ses-3) | -2.532 | 0.453 | 1430.53 | -5.584 | <0.001 | <0.001 |
| Scheduled C-Section | (ses-6) - (ses-3) | -2.856 | 0.422 | 1430.53 | -6.766 | <0.001 | <0.001 |
| Control | (ses-2) - (ses-1) | -0.138 | 0.220 | 1430.53 | -0.629 | 0.530 | 0.628 |
| Control | (ses-3) - (ses-1) | -0.179 | 0.217 | 1430.53 | -0.828 | 0.408 | 0.502 |
| Control | (ses-4) - (ses-1) | -0.113 | 0.206 | 1430.53 | -0.550 | 0.582 | 0.644 |
| Control | (ses-5) - (ses-1) | -0.125 | 0.228 | 1430.53 | -0.548 | 0.584 | 0.644 |
| Control | (ses-6) - (ses-1) | -0.248 | 0.249 | 1430.53 | -0.996 | 0.319 | 0.444 |
| Control | (ses-4) - (ses-3) | 0.066 | 0.189 | 1430.53 | 0.351 | 0.726 | 0.774 |
| Control | (ses-5) - (ses-3) | 0.055 | 0.220 | 1430.53 | 0.248 | 0.804 | 0.804 |
| Control | (ses-6) - (ses-3) | -0.069 | 0.226 | 1430.53 | -0.304 | 0.761 | 0.785 |

| Session | Contrast | Estimate | SE | df | t.ratio | p.value | pFDR |
|---|---|---|---|---|---|---|---|
| ses-1 | Vaginal - (Emergency CS) | 0.254 | 0.582 | 1430.53 | 0.436 | 0.663 | 0.979 |
| ses-1 | Vaginal - (Scheduled CS) | 0.926 | 0.786 | 1430.53 | 1.179 | 0.239 | 0.864 |
| ses-1 | Vaginal - Control | -0.250 | 0.442 | 1430.53 | -0.566 | 0.571 | 0.979 |
| ses-1 | (Emergency CS) - (Scheduled CS) | 0.672 | 0.896 | 1430.53 | 0.751 | 0.453 | 0.979 |
| ses-1 | (Emergency CS) - Control | -0.504 | 0.621 | 1430.53 | -0.812 | 0.417 | 0.979 |
| ses-1 | (Scheduled CS) - Control | -1.176 | 0.816 | 1430.53 | -1.441 | 0.150 | 0.674 |
| ses-2 | Vaginal - (Emergency CS) | 0.066 | 0.583 | 1430.53 | 0.113 | 0.910 | 0.979 |
| ses-2 | Vaginal - (Scheduled CS) | 0.421 | 0.785 | 1430.53 | 0.537 | 0.592 | 0.979 |
| ses-2 | Vaginal - Control | 0.452 | 0.447 | 1430.53 | 1.011 | 0.312 | 0.979 |
| ses-2 | (Emergency CS) - (Scheduled CS) | 0.356 | 0.897 | 1430.53 | 0.396 | 0.692 | 0.979 |
| ses-2 | (Emergency CS) - Control | 0.386 | 0.628 | 1430.53 | 0.615 | 0.539 | 0.979 |
| ses-2 | (Scheduled CS) - Control | 0.031 | 0.819 | 1430.53 | 0.037 | 0.970 | 0.990 |
| ses-3 | Vaginal - (Emergency CS) | -0.098 | 0.558 | 1430.53 | -0.176 | 0.860 | 0.979 |
| ses-3 | Vaginal - (Scheduled CS) | 0.105 | 0.737 | 1430.53 | 0.143 | 0.887 | 0.979 |
| ses-3 | Vaginal - Control | 2.377 | 0.425 | 1430.53 | 5.596 | <0.001 | <0.001 |
| ses-3 | (Emergency CS) - (Scheduled CS) | 0.203 | 0.852 | 1430.53 | 0.239 | 0.811 | 0.979 |
| ses-3 | (Emergency CS) - Control | 2.475 | 0.606 | 1430.53 | 4.082 | <0.001 | <0.001 |
| ses-3 | (Scheduled CS) - Control | 2.271 | 0.775 | 1430.53 | 2.930 | 0.003 | 0.031 |

| | | | | | | | |
|---|---|---|---|---|---|---|---|
| ses-4 | Vaginal - (Emergency CS) | 0.125 | 0.556 | 1430.53 | 0.226 | 0.821 | 0.979 |
| ses-4 | Vaginal - (Scheduled CS) | 0.301 | 0.735 | 1430.53 | 0.410 | 0.682 | 0.979 |
| ses-4 | Vaginal - Control | 1.474 | 0.420 | 1430.53 | 3.506 | <0.001 | 0.006 |
| ses-4 | (Emergency CS) - (Scheduled CS) | 0.176 | 0.849 | 1430.53 | 0.207 | 0.836 | 0.979 |
| ses-4 | (Emergency CS) - Control | 1.348 | 0.601 | 1430.53 | 2.242 | 0.025 | 0.181 |
| ses-4 | (Scheduled CS) - Control | 1.172 | 0.771 | 1430.53 | 1.520 | 0.129 | 0.662 |
| ses-5 | Vaginal - (Emergency CS) | 0.415 | 0.587 | 1430.53 | 0.707 | 0.479 | 0.979 |
| ses-5 | Vaginal - (Scheduled CS) | -0.009 | 0.786 | 1430.53 | -0.012 | 0.990 | 0.990 |
| ses-5 | Vaginal - Control | -0.325 | 0.447 | 1430.53 | -0.726 | 0.468 | 0.979 |
| ses-5 | (Emergency CS) - (Scheduled CS) | -0.425 | 0.899 | 1430.53 | -0.472 | 0.637 | 0.979 |
| ses-5 | (Emergency CS) - Control | -0.740 | 0.629 | 1430.53 | -1.175 | 0.240 | 0.864 |
| ses-5 | (Scheduled CS) - Control | -0.315 | 0.818 | 1430.53 | -0.385 | 0.700 | 0.979 |
| ses-6 | Vaginal - (Emergency CS) | -0.139 | 0.572 | 1430.53 | -0.242 | 0.809 | 0.979 |
| ses-6 | Vaginal - (Scheduled CS) | -0.221 | 0.764 | 1430.53 | -0.290 | 0.772 | 0.979 |
| ses-6 | Vaginal - Control | -0.737 | 0.442 | 1430.53 | -1.668 | 0.095 | 0.573 |
| ses-6 | (Emergency CS) - (Scheduled CS) | -0.083 | 0.876 | 1430.53 | -0.094 | 0.925 | 0.979 |
| ses-6 | (Emergency CS) - Control | -0.599 | 0.620 | 1430.53 | -0.966 | 0.334 | 0.979 |
| ses-6 | (Scheduled CS) - Control | -0.516 | 0.801 | 1430.53 | -0.644 | 0.519 | 0.979 |

| Sessions | Contrast | Estimate | SE | df | t.ratio | p.value | pFDR |
|---|---|---|---|---|---|---|---|
| (ses-2) - (ses-1) | (Emergency CS) - (Scheduled CS) | -0.317 | 0.573 | 1430.53 | -0.553 | 0.580 | 0.714 |
| (ses-2) - (ses-1) | (Emergency CS) - (Control) | 0.890 | 0.381 | 1430.53 | 2.339 | 0.019 | 0.055 |
| (ses-2) - (ses-1) | (Scheduled CS) - (Control) | 1.207 | 0.527 | 1430.53 | 2.289 | 0.022 | 0.059 |
| (ses-2) - (ses-1) | (Vaginal) - (Emergency CS) | -0.188 | 0.364 | 1430.53 | -0.516 | 0.606 | 0.723 |
| (ses-2) - (ses-1) | (Vaginal) - (Scheduled CS) | -0.505 | 0.516 | 1430.53 | -0.979 | 0.328 | 0.492 |
| (ses-2) - (ses-1) | (Vaginal) - (Control) | 0.702 | 0.288 | 1430.53 | 2.439 | 0.015 | 0.044 |
| (ses-3) - (ses-1) | (Emergency CS) - (Scheduled CS) | -0.469 | 0.542 | 1430.53 | -0.866 | 0.387 | 0.546 |
| (ses-3) - (ses-1) | (Emergency CS) - (Control) | 2.979 | 0.364 | 1430.53 | 8.187 | <0.001 | <0.001 |
| (ses-3) - (ses-1) | (Scheduled CS) - (Control) | 3.448 | 0.500 | 1430.53 | 6.901 | <0.001 | <0.001 |
| (ses-3) - (ses-1) | (Vaginal) - (Emergency CS) | -0.352 | 0.343 | 1430.53 | -1.025 | 0.305 | 0.473 |
| (ses-3) - (ses-1) | (Vaginal) - (Scheduled CS) | -0.821 | 0.485 | 1430.53 | -1.693 | 0.091 | 0.198 |
| (ses-3) - (ses-1) | (Vaginal) - (Control) | 2.627 | 0.273 | 1430.53 | 9.640 | <0.001 | <0.001 |
| (ses-4) - (ses-1) | (Emergency CS) - (Scheduled CS) | -0.496 | 0.535 | 1430.53 | -0.928 | 0.354 | 0.514 |
| (ses-4) - (ses-1) | (Emergency CS) - (Control) | 1.852 | 0.353 | 1430.53 | 5.246 | <0.001 | <0.001 |
| (ses-4) - (ses-1) | (Scheduled CS) - (Control) | 2.349 | 0.490 | 1430.53 | 4.793 | <0.001 | <0.001 |
| (ses-4) - (ses-1) | (Vaginal) - (Emergency CS) | -0.128 | 0.338 | 1430.53 | -0.379 | 0.705 | 0.787 |
| (ses-4) - (ses-1) | (Vaginal) - (Scheduled CS) | -0.625 | 0.480 | 1430.53 | -1.302 | 0.193 | 0.343 |
| (ses-4) - (ses-1) | (Vaginal) - (Control) | 1.724 | 0.262 | 1430.53 | 6.578 | <0.001 | <0.001 |
| (ses-5) - (ses-1) | (Emergency CS) - (Scheduled CS) | -1.097 | 0.580 | 1430.53 | -1.890 | 0.059 | 0.142 |
| (ses-5) - (ses-1) | (Emergency CS) - (Control) | -0.236 | 0.387 | 1430.53 | -0.610 | 0.542 | 0.703 |
| (ses-5) - (ses-1) | (Scheduled CS) - (Control) | 0.861 | 0.530 | 1430.53 | 1.625 | 0.104 | 0.209 |
| (ses-5) - (ses-1) | (Vaginal) - (Emergency CS) | 0.161 | 0.375 | 1430.53 | 0.430 | 0.667 | 0.762 |
| (ses-5) - (ses-1) | (Vaginal) - (Scheduled CS) | -0.936 | 0.522 | 1430.53 | -1.794 | 0.073 | 0.167 |
| (ses-5) - (ses-1) | (Vaginal) - (Control) | -0.074 | 0.291 | 1430.53 | -0.256 | 0.798 | 0.848 |
| (ses-6) - (ses-1) | (Emergency CS) - (Scheduled CS) | -0.755 | 0.564 | 1430.53 | -1.340 | 0.180 | 0.333 |
| (ses-6) - (ses-1) | (Emergency CS) - (Control) | -0.095 | 0.383 | 1430.53 | -0.247 | 0.805 | 0.848 |
| (ses-6) - (ses-1) | (Scheduled CS) - (Control) | 0.660 | 0.522 | 1430.53 | 1.265 | 0.206 | 0.354 |
| (ses-6) - (ses-1) | (Vaginal) - (Emergency CS) | -0.392 | 0.363 | 1430.53 | -1.080 | 0.280 | 0.449 |
| (ses-6) - (ses-1) | (Vaginal) - (Scheduled CS) | -1.147 | 0.508 | 1430.53 | -2.258 | 0.024 | 0.061 |
| (ses-6) - (ses-1) | (Vaginal) - (Control) | -0.487 | 0.295 | 1430.53 | -1.649 | 0.099 | 0.207 |
| (ses-4) - (ses-3) | (Emergency CS) - (Scheduled CS) | -0.027 | 0.453 | 1430.53 | -0.060 | 0.952 | 0.952 |
| (ses-4) - (ses-3) | (Emergency CS) - (Control) | -1.126 | 0.323 | 1430.53 | -3.490 | <0.001 | 0.002 |
| (ses-4) - (ses-3) | (Scheduled CS) - (Control) | -1.099 | 0.415 | 1430.53 | -2.649 | 0.008 | 0.026 |
| (ses-4) - (ses-3) | (Vaginal) - (Emergency CS) | 0.224 | 0.293 | 1430.53 | 0.762 | 0.446 | 0.612 |
| (ses-4) - (ses-3) | (Vaginal) - (Scheduled CS) | 0.196 | 0.392 | 1430.53 | 0.500 | 0.617 | 0.723 |

| | | | | | | | |
|---|---|---|---|---|---|---|---|
| (ses-4) - (ses-3) | (Vaginal) - (Control) | -0.903 | 0.230 | 1430.53 | -3.923 | <0.001 | <0.001 |
| (ses-5) - (ses-3) | (Emergency CS) - (Scheduled CS) | -0.628 | 0.545 | 1430.53 | -1.151 | 0.250 | 0.413 |
| (ses-5) - (ses-3) | (Emergency CS) - (Control) | -3.214 | 0.374 | 1430.53 | -8.584 | <0.001 | <0.001 |
| (ses-5) - (ses-3) | (Scheduled CS) - (Control) | -2.587 | 0.503 | 1430.53 | -5.145 | <0.001 | <0.001 |
| (ses-5) - (ses-3) | (Vaginal) - (Emergency CS) | 0.513 | 0.350 | 1430.53 | 1.465 | 0.143 | 0.275 |
| (ses-5) - (ses-3) | (Vaginal) - (Scheduled CS) | -0.115 | 0.485 | 1430.53 | -0.237 | 0.813 | 0.848 |
| (ses-5) - (ses-3) | (Vaginal) - (Control) | -2.701 | 0.279 | 1430.53 | -9.671 | <0.001 | <0.001 |
| (ses-6) - (ses-3) | (Emergency CS) - (Scheduled CS) | -0.286 | 0.504 | 1430.53 | -0.567 | 0.571 | 0.714 |
| (ses-6) - (ses-3) | (Emergency CS) - (Control) | -3.073 | 0.356 | 1430.53 | -8.626 | <0.001 | <0.001 |
| (ses-6) - (ses-3) | (Scheduled CS) - (Control) | -2.787 | 0.471 | 1430.53 | -5.917 | <0.001 | <0.001 |
| (ses-6) - (ses-3) | (Vaginal) - (Emergency CS) | -0.040 | 0.323 | 1430.53 | -0.125 | 0.901 | 0.920 |
| (ses-6) - (ses-3) | (Vaginal) - (Scheduled CS) | -0.326 | 0.446 | 1430.53 | -0.731 | 0.465 | 0.620 |
| (ses-6) - (ses-3) | (Vaginal) - (Control) | -3.114 | 0.268 | 1430.53 | -11.602 | <0.001 | <0.001 |

**Supplementary Table 9.** HGAM coefficients and smooth terms for the cohort dataset using *pyment.*

| Coefficient | Estimate | Std.Error | t.value | p.value |
|---|---|---|---|---|
| (Intercept) | -2.900 | 0.291 | -9.981 | <0.001 |
| Group [Non-Gestational Mother] | -0.969 | 0.574 | -1.686 | 0.092 |
| Group [Nulliparous Women] | -1.092 | 0.391 | -2.795 | 0.005 |
| Age | -0.160 | 0.060 | -2.651 | 0.008 |
| Dataset [Barcelona] | 0.373 | 0.339 | 1.098 | 0.272 |
| Age : Dataset [Barcelona] | -0.086 | 0.070 | -1.224 | 0.221 |

| Smooth Term | edf | Ref.df | F | p.value | p.FDR |
|---|---|---|---|---|---|
| s(Time to Parturition) : Group [Gestational Mother] | 7.906 | 8.581 | 136.930 | <0.001 | <0.001 |
| s(Time to Parturition) : Group [Non-Gestational Mother] | 1.001 | 1.002 | 0.088 | 0.768 | 0.997 |
| s(Time to Parturition) : Group [Nulliparous Women] | 1.001 | 1.001 | 0.000 | 0.997 | 0.997 |
| s(Participant) | 446.014 | 464.000 | 30.624 | <0.001 | - |

Deviance explained = 0.922, $R^2$(Adjusted) = 0.898, n = 1943

**Supplementary Table 10.** HGAM coefficients and smooth terms for the cohort dataset using *DeepBrainNet.*

| Coefficient | Estimate | Std.Error | t.value | p.value |
|---|---|---|---|---|
| (Intercept) | -2.604 | 0.525 | -4.963 | <0.001 |
| Group [Non-Gestational Mother] | -0.844 | 1.031 | -0.818 | 0.413 |
| Group [Nulliparous Women] | -3.144 | 0.702 | -4.476 | <0.001 |
| Age | -0.166 | 0.110 | -1.501 | 0.134 |
| Dataset [Barcelona] | 2.468 | 0.612 | 4.031 | <0.001 |
| Age : Dataset [Barcelona] | -0.184 | 0.130 | -1.420 | 0.156 |

| Smooth Term | edf | Ref.df | F | p.value | p.FDR |
|---|---|---|---|---|---|
| s(Time to Parturition) : Group [Gestational Mother] | 8.418 | 8.858 | 115.253 | <0.001 | <0.001 |
| s(Time to Parturition) : Group [Non-Gestational Mother] | 1.260 | 1.469 | 4.300 | 0.039 | 0.059 |
| s(Time to Parturition) : Group [Nulliparous Women] | 1.003 | 1.006 | 2.130 | 0.145 | 0.145 |
| s(Participant) | 441.879 | 464.000 | 23.464 | <0.001 | - |

Deviance explained = 0.904, $R^2$(Adjusted) = 0.874, n = 1943

**Supplementary Table 11.** HGAM coefficients and smooth terms for the cohort dataset using *brainAGE.*

| Coefficient | Estimate | Std.Error | t.value | p.value |
|---|---|---|---|---|
| (Intercept) | 3.555 | 0.378 | 9.405 | <0.001 |
| Group [Non-Gestational Mother] | -1.487 | 0.753 | -1.974 | 0.049 |
| Group [Nulliparous Women] | -1.689 | 0.511 | -3.305 | <0.001 |
| Age | -0.306 | 0.077 | -3.972 | <0.001 |
| Dataset [Barcelona] | -1.622 | 0.442 | -3.668 | <0.001 |
| Age : Dataset [Barcelona] | 0.077 | 0.089 | 0.870 | 0.385 |

| Smooth Term | edf | Ref.df | F | p.value | p.FDR |
|---|---|---|---|---|---|
| s(Time to Parturition) : Group [Gestational Mother] | 8.426 | 8.860 | 183.108 | <0.001 | <0.001 |
| s(Time to Parturition) : Group [Non-Gestational Mother] | 1.003 | 1.006 | 0.079 | 0.782 | 0.782 |
| s(Time to Parturition) : Group [Nulliparous Women] | 1.646 | 2.003 | 1.561 | 0.213 | 0.319 |
| s(Participant) | 452.503 | 464.000 | 53.256 | <0.001 | - |

Deviance explained = 0.954, $R^2$(Adjusted) = 0.940, n = 1943

**Supplementary Table 12.** LMEM brain age gap estimates of the cohort dataset derived from *pyment*.

| Coefficient | Estimate | Std.Error | t.value | p.value |
|---|---|---|---|---|
| (Intercept) | -4.249 | 0.309 | -13.746 | <0.001 |
| Group [Non-Gestational Mother] | 0.279 | 0.608 | 0.458 | 0.647 |
| Group [Nulliparous Women] | 0.184 | 0.428 | 0.431 | 0.667 |
| Session [ses-2] | 0.987 | 0.128 | 7.701 | <0.001 |
| Session [ses-3] | 2.556 | 0.126 | 20.272 | <0.001 |
| Session [ses-4] | 2.488 | 0.127 | 19.531 | <0.001 |
| Session [ses-5] | 0.503 | 0.146 | 3.441 | <0.001 |
| Session [ses-6] | -0.218 | 0.159 | -1.368 | 0.171 |
| Age | -0.212 | 0.057 | -3.696 | <0.001 |
| Dataset [Barcelona] | 0.422 | 0.338 | 1.247 | 0.213 |
| Group [Non-Gestational Mother] : Session [ses-2] | -0.838 | 0.305 | -2.751 | 0.006 |
| Group [Nulliparous Women] : Session [ses-2] | -0.924 | 0.273 | -3.385 | <0.001 |
| Group [Non-Gestational Mother] : Session [ses-3] | -2.866 | 0.314 | -9.122 | <0.001 |
| Group [Nulliparous Women] : Session [ses-3] | -2.456 | 0.251 | -9.776 | <0.001 |
| Group [Non-Gestational Mother] : Session [ses-4] | -2.229 | 0.304 | -7.336 | <0.001 |
| Group [Nulliparous Women] : Session [ses-4] | -2.433 | 0.237 | -10.257 | <0.001 |
| Group [Non-Gestational Mother] : Session [ses-5] | -0.417 | 0.322 | -1.296 | 0.195 |
| Group [Nulliparous Women] : Session [ses-5] | -0.426 | 0.263 | -1.622 | 0.105 |
| Group [Non-Gestational Mother] : Session [ses-6] | 0.309 | 0.357 | 0.864 | 0.388 |
| Group [Nulliparous Women] : Session [ses-6] | 0.202 | 0.264 | 0.766 | 0.444 |
| Age : Dataset [Barcelona] | -0.014 | 0.066 | -0.214 | 0.831 |

| Smooth | edf | Ref.df | F | p.value |
|---|---|---|---|---|
| s(Participant ID) | 446.00 | 464.00 | 30.228 | <0.001 |

Deviance explained = 0.921, $R^2$(Adjusted) = 0.896, n = 1943

| Group | Contrast | Estimate | SE | df | t.ratio | p.value | pFDR |
|---|---|---|---|---|---|---|---|
| Gestational Mother | (ses-2) - (ses-1) | 0.987 | 0.128 | 1476.00 | 7.701 | <0.001 | <0.001 |
| Gestational Mother | (ses-3) - (ses-1) | 2.556 | 0.126 | 1476.00 | 20.272 | <0.001 | <0.001 |
| Gestational Mother | (ses-4) - (ses-1) | 2.488 | 0.127 | 1476.00 | 19.531 | <0.001 | <0.001 |
| Gestational Mother | (ses-5) - (ses-1) | 0.503 | 0.146 | 1476.00 | 3.441 | <0.001 | 0.002 |
| Gestational Mother | (ses-6) - (ses-1) | -0.218 | 0.159 | 1476.00 | -1.368 | 0.171 | 0.514 |
| Gestational Mother | (ses-4) - (ses-3) | -0.068 | 0.093 | 1476.00 | -0.737 | 0.461 | 0.851 |
| Gestational Mother | (ses-5) - (ses-3) | -2.054 | 0.119 | 1476.00 | -17.248 | <0.001 | <0.001 |
| Gestational Mother | (ses-6) - (ses-3) | -2.774 | 0.120 | 1476.00 | -23.131 | <0.001 | <0.001 |
| Non-Gestational Mother | (ses-2) - (ses-1) | 0.150 | 0.280 | 1476.00 | 0.534 | 0.593 | 0.883 |
| Non-Gestational Mother | (ses-3) - (ses-1) | -0.310 | 0.295 | 1476.00 | -1.051 | 0.293 | 0.640 |
| Non-Gestational Mother | (ses-4) - (ses-1) | 0.259 | 0.285 | 1476.00 | 0.908 | 0.364 | 0.729 |
| Non-Gestational Mother | (ses-5) - (ses-1) | 0.085 | 0.303 | 1476.00 | 0.281 | 0.779 | 0.883 |
| Non-Gestational Mother | (ses-6) - (ses-1) | 0.091 | 0.349 | 1476.00 | 0.260 | 0.795 | 0.883 |
| Non-Gestational Mother | (ses-4) - (ses-3) | 0.569 | 0.291 | 1476.00 | 1.956 | 0.051 | 0.174 |
| Non-Gestational Mother | (ses-5) - (ses-3) | 0.395 | 0.306 | 1476.00 | 1.290 | 0.197 | 0.526 |
| Non-Gestational Mother | (ses-6) - (ses-3) | 0.401 | 0.349 | 1476.00 | 1.147 | 0.251 | 0.603 |
| Nulliparous Women | (ses-2) - (ses-1) | 0.064 | 0.244 | 1476.00 | 0.261 | 0.794 | 0.883 |
| Nulliparous Women | (ses-3) - (ses-1) | 0.101 | 0.228 | 1476.00 | 0.441 | 0.659 | 0.883 |
| Nulliparous Women | (ses-4) - (ses-1) | 0.055 | 0.213 | 1476.00 | 0.258 | 0.796 | 0.883 |
| Nulliparous Women | (ses-5) - (ses-1) | 0.076 | 0.237 | 1476.00 | 0.321 | 0.748 | 0.883 |
| Nulliparous Women | (ses-6) - (ses-1) | -0.015 | 0.249 | 1476.00 | -0.061 | 0.951 | 0.951 |
| Nulliparous Women | (ses-4) - (ses-3) | -0.046 | 0.189 | 1476.00 | -0.242 | 0.809 | 0.883 |
| Nulliparous Women | (ses-5) - (ses-3) | -0.024 | 0.232 | 1476.00 | -0.105 | 0.916 | 0.951 |
| Nulliparous Women | (ses-6) - (ses-3) | -0.116 | 0.222 | 1476.00 | -0.521 | 0.603 | 0.883 |

**Supplementary Table 13.** LMEM brain age gap estimates of the cohort dataset derived from *DeepBrainNet*.

| Coefficient | Estimate | Std.Error | t.value | p.value |
|---|---|---|---|---|
| (Intercept) | -4.522 | 0.567 | -7.968 | <0.001 |
| Group [Non-Gestational Mother] | 0.533 | 1.114 | 0.479 | 0.632 |
| Group [Nulliparous Women] | -1.040 | 0.791 | -1.315 | 0.189 |
| Session [ses-2] | 2.320 | 0.258 | 9.003 | <0.001 |
| Session [ses-3] | 4.889 | 0.251 | 19.460 | <0.001 |
| Session [ses-4] | 2.276 | 0.253 | 8.995 | <0.001 |
| Session [ses-5] | -0.305 | 0.289 | -1.054 | 0.292 |
| Session [ses-6] | -1.094 | 0.310 | -3.529 | <0.001 |
| Age | -0.331 | 0.106 | -3.119 | 0.002 |
| Dataset [Barcelona] | 2.685 | 0.613 | 4.379 | <0.001 |
| Group [Non-Gestational Mother] : Session [ses-2] | -2.267 | 0.616 | -3.681 | <0.001 |
| Group [Nulliparous Women] : Session [ses-2] | -2.498 | 0.552 | -4.526 | <0.001 |
| Group [Non-Gestational Mother] : Session [ses-3] | -5.246 | 0.635 | -8.258 | <0.001 |
| Group [Nulliparous Women] : Session [ses-3] | -4.955 | 0.507 | -9.763 | <0.001 |
| Group [Non-Gestational Mother] : Session [ses-4] | -2.192 | 0.614 | -3.568 | <0.001 |
| Group [Nulliparous Women] : Session [ses-4] | -2.715 | 0.479 | -5.665 | <0.001 |
| Group [Non-Gestational Mother] : Session [ses-5] | 1.342 | 0.651 | 2.060 | 0.040 |
| Group [Nulliparous Women] : Session [ses-5] | -0.212 | 0.531 | -0.400 | 0.689 |
| Group [Non-Gestational Mother] : Session [ses-6] | 2.265 | 0.722 | 3.136 | 0.002 |
| Group [Nulliparous Women] : Session [ses-6] | 0.451 | 0.534 | 0.846 | 0.398 |
| Age : Dataset [Barcelona] | 0.042 | 0.123 | 0.344 | 0.731 |

| Smooth | edf | Ref.df | F | p.value |
|---|---|---|---|---|

| | | | | |
|---|---|---|---|---|
| s(Participant ID) | 441.92 | 464.00 | 23.098 | <0.001 |

Deviance explained = 0.902, $R^2$(Adjusted) = 0.871, n = 1943

| Group | Contrast | Estimate | SE | df | t.ratio | p.value | pFDR |
|---|---|---|---|---|---|---|---|
| Gestational Mother | (ses-2) - (ses-1) | 2.320 | 0.258 | 1480.08 | 9.003 | <0.001 | <0.001 |
| Gestational Mother | (ses-3) - (ses-1) | 4.889 | 0.251 | 1480.08 | 19.460 | <0.001 | <0.001 |
| Gestational Mother | (ses-4) - (ses-1) | 2.276 | 0.253 | 1480.08 | 8.995 | <0.001 | <0.001 |
| Gestational Mother | (ses-5) - (ses-1) | -0.305 | 0.289 | 1480.08 | -1.054 | 0.292 | 0.446 |
| Gestational Mother | (ses-6) - (ses-1) | -1.094 | 0.310 | 1480.08 | -3.529 | <0.001 | 0.001 |
| Gestational Mother | (ses-4) - (ses-3) | -2.613 | 0.188 | 1480.08 | -13.918 | <0.001 | <0.001 |
| Gestational Mother | (ses-5) - (ses-3) | -5.194 | 0.240 | 1480.08 | -21.643 | <0.001 | <0.001 |
| Gestational Mother | (ses-6) - (ses-3) | -5.982 | 0.238 | 1480.08 | -25.149 | <0.001 | <0.001 |
| Non-Gestational Mother | (ses-2) - (ses-1) | 0.053 | 0.565 | 1480.08 | 0.093 | 0.926 | 0.926 |
| Non-Gestational Mother | (ses-3) - (ses-1) | -0.357 | 0.595 | 1480.08 | -0.600 | 0.549 | 0.658 |
| Non-Gestational Mother | (ses-4) - (ses-1) | 0.084 | 0.575 | 1480.08 | 0.146 | 0.884 | 0.925 |
| Non-Gestational Mother | (ses-5) - (ses-1) | 1.037 | 0.610 | 1480.08 | 1.701 | 0.089 | 0.206 |
| Non-Gestational Mother | (ses-6) - (ses-1) | 1.171 | 0.700 | 1480.08 | 1.674 | 0.094 | 0.206 |
| Non-Gestational Mother | (ses-4) - (ses-3) | 0.441 | 0.588 | 1480.08 | 0.750 | 0.453 | 0.573 |
| Non-Gestational Mother | (ses-5) - (ses-3) | 1.394 | 0.619 | 1480.08 | 2.253 | 0.024 | 0.073 |
| Non-Gestational Mother | (ses-6) - (ses-3) | 1.528 | 0.704 | 1480.08 | 2.171 | 0.030 | 0.080 |
| Nulliparous Women | (ses-2) - (ses-1) | -0.178 | 0.493 | 1480.08 | -0.360 | 0.719 | 0.821 |
| Nulliparous Women | (ses-3) - (ses-1) | -0.066 | 0.459 | 1480.08 | -0.143 | 0.886 | 0.925 |
| Nulliparous Women | (ses-4) - (ses-1) | -0.439 | 0.429 | 1480.08 | -1.024 | 0.306 | 0.446 |
| Nulliparous Women | (ses-5) - (ses-1) | -0.517 | 0.477 | 1480.08 | -1.084 | 0.279 | 0.446 |
| Nulliparous Women | (ses-6) - (ses-1) | -0.642 | 0.497 | 1480.08 | -1.292 | 0.197 | 0.364 |
| Nulliparous Women | (ses-4) - (ses-3) | -0.374 | 0.381 | 1480.08 | -0.980 | 0.327 | 0.446 |
| Nulliparous Women | (ses-5) - (ses-3) | -0.451 | 0.468 | 1480.08 | -0.965 | 0.335 | 0.446 |
| Nulliparous Women | (ses-6) - (ses-3) | -0.577 | 0.447 | 1480.08 | -1.290 | 0.197 | 0.364 |

**Supplementary Table 14.** LMEM brain age gap estimates of the cohort dataset derived from *BrainAGE*.

| Coefficient | Estimate | Std.Error | t.value | p.value |
|---|---|---|---|---|
| (Intercept) | 2.589 | 0.395 | 6.561 | <0.001 |
| Group [Non-Gestational Mother] | -0.258 | 0.780 | -0.330 | 0.741 |
| Group [Nulliparous Women] | -0.278 | 0.540 | -0.514 | 0.608 |
| Session [ses-2] | 0.449 | 0.132 | 3.404 | <0.001 |
| Session [ses-3] | 2.899 | 0.133 | 21.795 | <0.001 |
| Session [ses-4] | 2.013 | 0.136 | 14.854 | <0.001 |
| Session [ses-5] | -0.427 | 0.157 | -2.717 | 0.007 |
| Session [ses-6] | -0.884 | 0.178 | -4.960 | <0.001 |
| Age | -0.382 | 0.071 | -5.402 | <0.001 |
| Dataset [Barcelona] | -1.640 | 0.441 | -3.722 | <0.001 |
| Group [Non-Gestational Mother] : Session [ses-2] | -0.929 | 0.308 | -3.017 | 0.003 |
| Group [Nulliparous Women] : Session [ses-2] | -0.920 | 0.276 | -3.334 | <0.001 |
| Group [Non-Gestational Mother] : Session [ses-3] | -3.429 | 0.318 | -10.794 | <0.001 |
| Group [Nulliparous Women] : Session [ses-3] | -3.445 | 0.254 | -13.547 | <0.001 |
| Group [Non-Gestational Mother] : Session [ses-4] | -2.033 | 0.307 | -6.621 | <0.001 |
| Group [Nulliparous Women] : Session [ses-4] | -2.558 | 0.240 | -10.659 | <0.001 |
| Group [Non-Gestational Mother] : Session [ses-5] | 0.313 | 0.326 | 0.961 | 0.337 |
| Group [Nulliparous Women] : Session [ses-5] | 0.036 | 0.266 | 0.137 | 0.891 |
| Group [Non-Gestational Mother] : Session [ses-6] | 0.546 | 0.361 | 1.510 | 0.131 |
| Group [Nulliparous Women] : Session [ses-6] | 0.345 | 0.267 | 1.291 | 0.197 |
| Age : Dataset [Barcelona] | 0.164 | 0.079 | 2.079 | 0.038 |

| Smooth | edf | Ref.df | F | p.value |
|---|---|---|---|---|
| s(Participant ID) | 452.71 | 464.00 | 52.986 | <0.001 |

Deviance explained = 0.954, $R^2$(Adjusted) = 0.939, n = 1943

| Group | Contrast | Estimate | SE | df | t.ratio | p.value | pFDR |
|---|---|---|---|---|---|---|---|
| Gestational Mother | (ses-2) - (ses-1) | 0.449 | 0.132 | 1469.29 | 3.404 | <0.001 | 0.002 |
| Gestational Mother | (ses-3) - (ses-1) | 2.899 | 0.133 | 1469.29 | 21.795 | <0.001 | <0.001 |
| Gestational Mother | (ses-4) - (ses-1) | 2.013 | 0.136 | 1469.29 | 14.854 | <0.001 | <0.001 |
| Gestational Mother | (ses-5) - (ses-1) | -0.427 | 0.157 | 1469.29 | -2.717 | 0.007 | 0.020 |
| Gestational Mother | (ses-6) - (ses-1) | -0.884 | 0.178 | 1469.29 | -4.960 | <0.001 | <0.001 |
| Gestational Mother | (ses-4) - (ses-3) | -0.886 | 0.094 | 1469.29 | -9.427 | <0.001 | <0.001 |
| Gestational Mother | (ses-5) - (ses-3) | -3.326 | 0.122 | 1469.29 | -27.346 | <0.001 | <0.001 |
| Gestational Mother | (ses-6) - (ses-3) | -3.783 | 0.128 | 1469.29 | -29.461 | <0.001 | <0.001 |
| Non-Gestational Mother | (ses-2) - (ses-1) | -0.479 | 0.284 | 1469.29 | -1.690 | 0.091 | 0.146 |
| Non-Gestational Mother | (ses-3) - (ses-1) | -0.530 | 0.300 | 1469.29 | -1.769 | 0.077 | 0.142 |
| Non-Gestational Mother | (ses-4) - (ses-1) | -0.020 | 0.291 | 1469.29 | -0.070 | 0.944 | 0.995 |
| Non-Gestational Mother | (ses-5) - (ses-1) | -0.114 | 0.310 | 1469.29 | -0.369 | 0.712 | 0.814 |
| Non-Gestational Mother | (ses-6) - (ses-1) | -0.339 | 0.361 | 1469.29 | -0.938 | 0.349 | 0.465 |
| Non-Gestational Mother | (ses-4) - (ses-3) | 0.510 | 0.294 | 1469.29 | 1.734 | 0.083 | 0.142 |
| Non-Gestational Mother | (ses-5) - (ses-3) | 0.416 | 0.310 | 1469.29 | 1.340 | 0.180 | 0.255 |
| Non-Gestational Mother | (ses-6) - (ses-3) | 0.191 | 0.356 | 1469.29 | 0.538 | 0.591 | 0.709 |
| Nulliparous Women | (ses-2) - (ses-1) | -0.471 | 0.248 | 1469.29 | -1.901 | 0.058 | 0.115 |
| Nulliparous Women | (ses-3) - (ses-1) | -0.547 | 0.233 | 1469.29 | -2.349 | 0.019 | 0.045 |
| Nulliparous Women | (ses-4) - (ses-1) | -0.545 | 0.218 | 1469.29 | -2.498 | 0.013 | 0.034 |
| Nulliparous Women | (ses-5) - (ses-1) | -0.391 | 0.245 | 1469.29 | -1.598 | 0.110 | 0.165 |
| Nulliparous Women | (ses-6) - (ses-1) | -0.539 | 0.262 | 1469.29 | -2.061 | 0.040 | 0.086 |
| Nulliparous Women | (ses-4) - (ses-3) | 0.001 | 0.191 | 1469.29 | 0.006 | 0.995 | 0.995 |
| Nulliparous Women | (ses-5) - (ses-3) | 0.156 | 0.235 | 1469.29 | 0.662 | 0.508 | 0.642 |
| Nulliparous Women | (ses-6) - (ses-3) | 0.007 | 0.229 | 1469.29 | 0.032 | 0.974 | 0.995 |

**Supplementary Table 15.** HGAM coefficients and smooth terms for the dense-sampling dataset using *pyment*, *DeepBrainNet* and *brainAGE*.

| Brain age Model | Coefficient | Estimate | Std.Error | t.value | p.value |
|---|---|---|---|---|---|
| pyment | (Intercept) | -5.093 | 0.244 | -20.868 | <0.001 |
| pyment | Scanning Site [UCSB] | -0.237 | 0.301 | -0.787 | 0.434 |
| DeepBrainNet | (Intercept) | -0.148 | 0.915 | -0.162 | 0.872 |
| DeepBrainNet | Scanning Site [UCSB] | -0.970 | 0.738 | -1.316 | 0.194 |
| brainAGE | (Intercept) | -2.010 | 0.755 | -2.662 | 0.010 |
| brainAGE | Scanning Site [UCSB] | 1.810 | 0.461 | 3.929 | <0.001 |

| Brain age Model | Smooth Term | edf | Ref.df | F | p.value | p.FDR |
|---|---|---|---|---|---|---|
| pyment | s(Time to Parturition) | 4.88 | 5.83 | 9.393 | <0.001 | <0.001 |
| pyment | s(Participant) | 0.00 | 2.00 | 0.000 | 0.579 | |
| DeepBrainNet | s(Time to Parturition) | 5.77 | 6.74 | 15.103 | <0.001 | <0.001 |
| DeepBrainNet | s(Participant) | 1.72 | 2.00 | 5.597 | 0.002 | |
| brainAGE | s(Time to Parturition) | 5.21 | 6.16 | 9.565 | <0.001 | <0.001 |
| brainAGE | s(Participant) | 1.87 | 2.00 | 11.456 | <0.001 | |

| Brain age Model | R-squared | Deviance Explained | N |
|---|---|---|---|
| pyment | 0.487 | 0.536 | 63 |
| DeepBrainNet | 0.750 | 0.784 | 63 |
| brainAGE | 0.523 | 0.585 | 63 |

**Supplementary Table 16.** LMEM brain age gap estimates of the dense-sampling dataset derived from *pyment, DeepBrainNet* and *BrainAge*.

| Brain age Model | Coefficient | Estimate | Std.Error | t.value | p.value |
|---|---|---|---|---|---|
| pyment | (Intercept) | -6.160 | 0.497 | -12.405 | <0.001 |
| pyment | Gestation/Postpartum [1st Trimester] | 0.706 | 0.495 | 1.427 | 0.159 |
| pyment | Gestation/Postpartum [2nd Trimester] | 1.386 | 0.489 | 2.835 | 0.006 |
| pyment | Gestation/Postpartum [3rd Trimester] | 2.352 | 0.547 | 4.296 | <0.001 |
| pyment | Gestation/Postpartum [Early Postpartum] | 3.061 | 0.665 | 4.601 | <0.001 |
| pyment | Gestation/Postpartum [~6 Months Postpartum] | 0.474 | 0.507 | 0.934 | 0.354 |
| pyment | Gestation/Postpartum [>1 Year Postpartum] | -1.277 | 0.617 | -2.070 | 0.043 |
| pyment | Scanning Site [UCSB] | -0.105 | 0.304 | -0.345 | 0.732 |
| DeepBrainNet | (Intercept) | -3.381 | 1.292 | -2.617 | 0.012 |
| DeepBrainNet | Gestation/Postpartum [1st Trimester] | 2.120 | 0.767 | 2.764 | 0.008 |
| DeepBrainNet | Gestation/Postpartum [2nd Trimester] | 3.993 | 0.796 | 5.013 | <0.001 |
| DeepBrainNet | Gestation/Postpartum [3rd Trimester] | 6.005 | 0.866 | 6.938 | <0.001 |
| DeepBrainNet | Gestation/Postpartum [Early Postpartum] | 5.546 | 1.023 | 5.421 | <0.001 |
| DeepBrainNet | Gestation/Postpartum [~6 Months Postpartum] | 2.371 | 0.821 | 2.889 | 0.006 |
| DeepBrainNet | Gestation/Postpartum [>1 Year Postpartum] | -0.203 | 0.918 | -0.221 | 0.826 |
| DeepBrainNet | Scanning Site [UCSB] | -0.612 | 0.758 | -0.807 | 0.423 |
| brainAGE | (Intercept) | -2.885 | 0.923 | -3.125 | 0.003 |
| brainAGE | Gestation/Postpartum [1st Trimester] | 0.707 | 0.478 | 1.480 | 0.145 |
| brainAGE | Gestation/Postpartum [2nd Trimester] | 1.015 | 0.498 | 2.040 | 0.046 |
| brainAGE | Gestation/Postpartum [3rd Trimester] | 2.126 | 0.540 | 3.936 | <0.001 |
| brainAGE | Gestation/Postpartum [Early Postpartum] | 2.477 | 0.637 | 3.888 | <0.001 |
| brainAGE | Gestation/Postpartum [~6 Months Postpartum] | 0.739 | 0.513 | 1.440 | 0.156 |
| brainAGE | Gestation/Postpartum [>1 Year Postpartum] | -1.085 | 0.570 | -1.903 | 0.062 |
| brainAGE | Scanning Site [UCSB] | 1.812 | 0.484 | 3.744 | <0.001 |

| Brain age Model | Smooth | edf | Ref.df | F | p.value |
|---|---|---|---|---|---|
| pyment | s(Participant ID) | 0.04 | 2.00 | 0.018 | 0.349 |
| DeepBrainNet | s(Participant ID) | 1.75 | 2.00 | 5.448 | 0.004 |
| brainAGE | s(Participant ID) | 1.86 | 2.00 | 9.571 | <0.001 |

| Brain age Model | R-squared | Deviance Explained | N |
|---|---|---|---|
| pyment | 0.502 | 0.559 | 63 |
| DeepBrainNet | 0.726 | 0.764 | 63 |
| brainAGE | 0.467 | 0.543 | 63 |

| Brain age Model | Contrast | Estimate | SE | df | t.ratio | p.value | pFDR |
|---|---|---|---|---|---|---|---|
| pyment | 1st Trimester - Pre-conception | 0.706 | 0.495 | 54.96 | 1.427 | 0.159 | 0.205 |

| | | | | | | | |
|---|---|---|---|---|---|---|---|
| pyment | 2nd Trimester - Pre-conception | 1.386 | 0.489 | 54.96 | 2.835 | 0.006 | 0.012 |
| pyment | 3rd Trimester - Pre-conception | 2.352 | 0.547 | 54.96 | 4.296 | <0.001 | <0.001 |
| pyment | Early Postpartum - Pre-conception | 3.061 | 0.665 | 54.96 | 4.601 | <0.001 | <0.001 |
| pyment | 6 Months Postpartum - Pre-conception | 0.474 | 0.507 | 54.96 | 0.934 | 0.354 | 0.354 |
| pyment | 1 Year Postpartum - Pre-conception | -1.277 | 0.617 | 54.96 | -2.070 | 0.043 | 0.065 |
| pyment | Early Postpartum - 3rd Trimester | 0.709 | 0.621 | 54.96 | 1.142 | 0.259 | 0.291 |
| pyment | 6 Months Postpartum - 3rd Trimester | -1.878 | 0.472 | 54.96 | -3.979 | <0.001 | <0.001 |
| pyment | 1 Year Postpartum - 3rd Trimester | -3.629 | 0.578 | 54.96 | -6.276 | <0.001 | <0.001 |
| DeepBrainNet | 1st Trimester - Pre-conception | 2.120 | 0.767 | 53.25 | 2.764 | 0.008 | 0.010 |
| DeepBrainNet | 2nd Trimester - Pre-conception | 3.993 | 0.796 | 53.25 | 5.013 | <0.001 | <0.001 |
| DeepBrainNet | 3rd Trimester - Pre-conception | 6.005 | 0.866 | 53.25 | 6.938 | <0.001 | <0.001 |
| DeepBrainNet | Early Postpartum - Pre-conception | 5.546 | 1.023 | 53.25 | 5.421 | <0.001 | <0.001 |
| DeepBrainNet | 6 Months Postpartum - Pre-conception | 2.371 | 0.821 | 53.25 | 2.889 | 0.006 | 0.008 |
| DeepBrainNet | 1 Year Postpartum - Pre-conception | -0.203 | 0.918 | 53.25 | -0.221 | 0.826 | 0.826 |
| DeepBrainNet | Early Postpartum - 3rd Trimester | -0.459 | 0.894 | 53.25 | -0.514 | 0.610 | 0.686 |
| DeepBrainNet | 6 Months Postpartum - 3rd Trimester | -3.634 | 0.680 | 53.25 | -5.343 | <0.001 | <0.001 |
| DeepBrainNet | 1 Year Postpartum - 3rd Trimester | -6.209 | 0.842 | 53.25 | -7.377 | <0.001 | <0.001 |
| brainAGE | 1st Trimester - Pre-conception | 0.707 | 0.478 | 53.14 | 1.480 | 0.145 | 0.175 |
| brainAGE | 2nd Trimester - Pre-conception | 1.015 | 0.498 | 53.14 | 2.040 | 0.046 | 0.083 |
| brainAGE | 3rd Trimester - Pre-conception | 2.126 | 0.540 | 53.14 | 3.936 | <0.001 | <0.001 |
| brainAGE | Early Postpartum - Pre-conception | 2.477 | 0.637 | 53.14 | 3.888 | <0.001 | <0.001 |
| brainAGE | 6 Months Postpartum - Pre-conception | 0.739 | 0.513 | 53.14 | 1.440 | 0.156 | 0.175 |
| brainAGE | 1 Year Postpartum - Pre-conception | -1.085 | 0.570 | 53.14 | -1.903 | 0.062 | 0.094 |
| brainAGE | Early Postpartum - 3rd Trimester | 0.351 | 0.553 | 53.14 | 0.634 | 0.529 | 0.529 |
| brainAGE | 6 Months Postpartum - 3rd Trimester | -1.387 | 0.421 | 53.14 | -3.294 | 0.002 | 0.004 |
| brainAGE | 1 Year Postpartum - 3rd Trimester | -3.211 | 0.522 | 53.14 | -6.155 | <0.001 | <0.001 |

**Supplementary Table 17.** Summary of GAM fits for brain age gap metrics, DNAm age and aging pace metrics.

| Metric | Temporal Smooth edf | Temporal Smooth Ref.df | Temporal Smooth F-statistic | Temporal Smooth p-value | Model $R^2$(Adjusted) | N |
|---|---|---|---|---|---|---|
| Brainage-gap [brainageR] | 5.83 | 6.78 | 12.016 | <0.001 | 0.758 | 27 |
| Brainage-gap [pyment] | 2.90 | 3.50 | 5.586 | 0.004 | 0.412 | 27 |
| Brainage-gap [DeepBrainNet] | 4.20 | 4.99 | 11.456 | <0.001 | 0.685 | 27 |
| Brainage-gap [brainAGE] | 5.01 | 5.91 | 6.367 | <0.001 | 0.589 | 27 |
| Age-gap [PCHorvath1] | 4.15 | 4.97 | 6.909 | 0.002 | 0.652 | 19 |
| Age-gap [PCHorvath2] | 4.11 | 4.92 | 7.188 | 0.002 | 0.659 | 19 |
| Age-gap [PCPhenoAge] | 4.78 | 5.66 | 10.511 | <0.001 | 0.764 | 19 |
| Age-gap [PCGrimAge] | 4.90 | 5.79 | 14.891 | <0.001 | 0.826 | 19 |
| Age-gap [DNAmGrimAge2] | 5.60 | 6.55 | 25.387 | <0.001 | 0.902 | 19 |
| DunedinPACE | 6.28 | 7.29 | 45.650 | <0.001 | 0.949 | 19 |

**Supplementary Table 18.** Pair-wise correlations between brain age gap metrics, age gap epigenetic clock metrics, and DunedinPACE.

| Metric 1 | Metric 2 | R | df | p-value | pFDR |
|---|---|---|---|---|---|
| Age-gap PCHorvath1 | Age-gap PCHorvath2 | 0.977 | 18 | <0.001 | <0.001 |
| Age-gap PCHorvath1 | Age-gap PCPhenoAge | 0.930 | 18 | <0.001 | <0.001 |
| Age-gap PCHorvath1 | Age-gap PCGrimAge | 0.895 | 18 | <0.001 | <0.001 |
| Age-gap PCHorvath1 | Age-gap DNAmGrimAge2 | 0.922 | 18 | <0.001 | <0.001 |

| | | | | | |
|---|---|---|---|---|---|
| Age-gap PCHorvath1 | DunedinPACE | 0.822 | 18 | <0.001 | <0.001 |
| Age-gap PCHorvath1 | Brainage-gap pyment | 0.312 | 17 | 0.193 | 0.263 |
| Age-gap PCHorvath1 | Brainage-gap brainageR | 0.641 | 17 | 0.003 | 0.006 |
| Age-gap PCHorvath1 | Brainage-gap DeepBrainNet | 0.561 | 17 | 0.013 | 0.019 |
| Age-gap PCHorvath1 | Brainage-gap brainAGE | -0.003 | 17 | 0.991 | 0.991 |
| Age-gap PCHorvath2 | Age-gap PCPhenoAge | 0.963 | 18 | <0.001 | <0.001 |
| Age-gap PCHorvath2 | Age-gap PCGrimAge | 0.934 | 18 | <0.001 | <0.001 |
| Age-gap PCHorvath2 | Age-gap DNAmGrimAge2 | 0.931 | 18 | <0.001 | <0.001 |
| Age-gap PCHorvath2 | DunedinPACE | 0.823 | 18 | <0.001 | <0.001 |
| Age-gap PCHorvath2 | Brainage-gap pyment | 0.254 | 17 | 0.293 | 0.380 |
| Age-gap PCHorvath2 | Brainage-gap brainageR | 0.590 | 17 | 0.008 | 0.013 |
| Age-gap PCHorvath2 | Brainage-gap DeepBrainNet | 0.583 | 17 | 0.009 | 0.014 |
| Age-gap PCHorvath2 | Brainage-gap brainAGE | -0.017 | 17 | 0.945 | 0.987 |
| Age-gap PCPhenoAge | Age-gap PCGrimAge | 0.981 | 18 | <0.001 | <0.001 |
| Age-gap PCPhenoAge | Age-gap DNAmGrimAge2 | 0.949 | 18 | <0.001 | <0.001 |
| Age-gap PCPhenoAge | DunedinPACE | 0.868 | 18 | <0.001 | <0.001 |
| Age-gap PCPhenoAge | Brainage-gap pyment | 0.144 | 17 | 0.557 | 0.660 |
| Age-gap PCPhenoAge | Brainage-gap brainageR | 0.613 | 17 | 0.005 | 0.009 |
| Age-gap PCPhenoAge | Brainage-gap DeepBrainNet | 0.583 | 17 | 0.009 | 0.014 |
| Age-gap PCPhenoAge | Brainage-gap brainAGE | 0.045 | 17 | 0.855 | 0.916 |
| Age-gap PCGrimAge | Age-gap DNAmGrimAge2 | 0.968 | 18 | <0.001 | <0.001 |
| Age-gap PCGrimAge | DunedinPACE | 0.895 | 18 | <0.001 | <0.001 |
| Age-gap PCGrimAge | Brainage-gap pyment | 0.120 | 17 | 0.624 | 0.720 |
| Age-gap PCGrimAge | Brainage-gap brainageR | 0.649 | 17 | 0.003 | 0.005 |
| Age-gap PCGrimAge | Brainage-gap DeepBrainNet | 0.653 | 17 | 0.002 | 0.005 |
| Age-gap PCGrimAge | Brainage-gap brainAGE | 0.061 | 17 | 0.804 | 0.905 |
| Age-gap DNAmGrimAge2 | DunedinPACE | 0.923 | 18 | <0.001 | <0.001 |
| Age-gap DNAmGrimAge2 | Brainage-gap pyment | 0.253 | 17 | 0.296 | 0.380 |
| Age-gap DNAmGrimAge2 | Brainage-gap brainageR | 0.711 | 17 | <0.001 | 0.001 |
| Age-gap DNAmGrimAge2 | Brainage-gap DeepBrainNet | 0.739 | 17 | <0.001 | <0.001 |
| Age-gap DNAmGrimAge2 | Brainage-gap brainAGE | -0.011 | 17 | 0.965 | 0.987 |
| DunedinPACE | Brainage-gap pyment | 0.249 | 17 | 0.304 | 0.380 |
| DunedinPACE | Brainage-gap brainageR | 0.726 | 17 | <0.001 | 0.001 |
| DunedinPACE | Brainage-gap DeepBrainNet | 0.739 | 17 | <0.001 | <0.001 |
| DunedinPACE | Brainage-gap brainAGE | -0.046 | 17 | 0.853 | 0.896 |
| Brainage-gap pyment | Brainage-gap brainageR | 0.568 | 25 | 0.002 | 0.004 |
| Brainage-gap pyment | Brainage-gap DeepBrainNet | 0.622 | 25 | <0.001 | 0.001 |
| Brainage-gap pyment | Brainage-gap brainAGE | 0.468 | 25 | 0.014 | 0.020 |
| Brainage-gap brainageR | Brainage-gap DeepBrainNet | 0.770 | 25 | <0.001 | <0.001 |
| Brainage-gap brainageR | Brainage-gap brainAGE | 0.387 | 25 | 0.046 | 0.065 |
| Brainage-gap DeepBrainNet | Brainage-gap brainAGE | 0.192 | 25 | 0.337 | 0.410 |

**Supplementary Table 19. Performance metrics of brain-age models**

| Brain age Model | Mean BAG (years) | SD BAG (years) | MAE (years) | r | r (BAG) | test-retest Adj. MAD (years) |
|---|---|---|---|---|---|---|
| brainAGE | 1.241 | 4.832 | 4.087 | 0.536 | -0.222 | 1.002 |
| brainageR | -6.431 | 4.273 | 6.573 | 0.320 | -0.716 | 0.736 |
| DeepBrainNet | -3.122 | 6.589 | 5.942 | 0.321 | -0.294 | 1.394 |
| pyment | -3.208 | 4.037 | 4.174 | 0.576 | -0.348 | 1.113 |

Metrics computed in the nulliparous women group of the cohort dataset ($N_{sessions}$ = 367). BAG=Brain age gap; SD=Standard deviation; MAE=mean absolute error (predicted brain-age vs chronological age); r=Pearson correlation coefficient of predicted brain age and chronological age; r BAG=Pearson correlation coefficient of brain age gap and chronological age. The test-retest Adj. MAD metric was computed using sessions 3 and 4 in the nulliparous women group of the cohort dataset (N = 142). Time elapsed between session 4 and session 3 was 0.168 ± 0.062 years (mean ± SD). Adj MAD=age-adjusted mean absolute difference of predicted brain-age between session 4 and session 3.

## References

1. Ginther, S. C., Cameron, H., White, C. R. & Marshall, D. J. Metabolic loads and the costs of metazoan reproduction. *Science* **384**, 763–767 (2024).
2. Giller, A., Andrawus, M., Gutman, D. & Atzmon, G. Pregnancy as a model for aging. *Ageing Research Reviews* **62**, 101093 (2020).
3. Pham, H. *et al.* The effects of pregnancy, its progression, and its cessation on human (maternal) biological aging. *Cell Metabolism* **36**, 877–878 (2024).
4. Poganik, J. R. *et al.* Biological age is increased by stress and restored upon recovery. *Cell Metabolism* **35**, 807-820.e5 (2023).
5. Gruzieva, O. *et al.* DNA Methylation Trajectories During Pregnancy. *Genet Epigenet* **12**, 2516865719867090 (2019).
6. Hoekzema, E. *et al.* Pregnancy leads to long-lasting changes in human brain structure. *Nature Neuroscience* **20**, 287–296 (2017).
7. Servin-Barthet, C. *et al.* Pregnancy entails a U-shaped trajectory in human brain structure linked to hormones and maternal attachment. *Nat Commun* **16**, 730 (2025).
8. Paternina-Die, M. *et al.* Women's neuroplasticity during gestation, childbirth and postpartum. *Nat Neurosci* **27**, 319–327 (2024).
9. Pritschet, L. *et al.* Neuroanatomical changes observed over the course of a human pregnancy. *Nat Neurosci* 1–8 (2024) doi:10.1038/s41593-024-01741-0.
10. Luders, E. *et al.* Potential Brain Age Reversal after Pregnancy: Younger Brains at 4–6 Weeks Postpartum. *Neuroscience* **386**, 309–314 (2018).
11. de Lange, A.-M. G. *et al.* The maternal brain: Region-specific patterns of brain aging are traceable decades after childbirth. *Hum Brain Mapp* **41**, 4718–4729 (2020).

12. de Lange, A.-M. G. *et al.* Population-based neuroimaging reveals traces of childbirth in the maternal brain. *Proc Natl Acad Sci USA* **116**, 22341–22346 (2019).
13. De Lange, A.-M. G. *et al.* Parental status and markers of brain and cellular age: A 3D convolutional network and classification study. *Psychoneuroendocrinology* 107040 (2024) doi:10.1016/j.psyneuen.2024.107040.
14. Dörfel, R. P. *et al.* Prediction of brain age using structural magnetic resonance imaging: A comparison of accuracy and test–retest reliability of publicly available software packages. *Human Brain Mapping* **44**, 6139–6148 (2023).
15. Lu, A. T. *et al.* DNA methylation GrimAge version 2. *Aging* https://doi.org/10.18632/aging.204434 (2022) doi:10.18632/aging.204434.
16. Belsky, D. W. *et al.* DunedinPACE, a DNA methylation biomarker of the pace of aging. *eLife* **11**, e73420 (2022).
17. Bethlehem, R. A. I. *et al.* Brain charts for the human lifespan. *Nature* **604**, 525–533 (2022).
18. Martínez-García, M. *et al.* Do Pregnancy-Induced Brain Changes Reverse? The Brain of a Mother Six Years after Parturition. *Brain sciences* **11**, 168 (2021).
19. Drewelies, J. *et al.* There Are Multiple Clocks That Time Us: Cross-Sectional and Longitudinal Associations Among 14 Alternative Indicators of Age and Aging. *The Journals of Gerontology, Series A: Biological Sciences and Medical Sciences* **80**, glae244 (2025).
20. Guintivano, J., Arad, M., Gould, T. D., Payne, J. L. & Kaminsky, Z. A. Antenatal prediction of postpartum depression with blood DNA methylation biomarkers. *Mol Psychiatry* **19**, 560–567 (2014).
21. Smew, A. I. *et al.* Limited association between markers of stress during pregnancy and fetal growth in 'Born into Life', a new prospective birth cohort. *Acta Paediatrica* **107**, 1003–1010 (2018).
22. Knight, A. K. *et al.* *SLC9B1* methylation predicts fetal intolerance of labor. *Epigenetics* **13**, 33–39 (2018).

23. White, W. M. *et al.* Normal early pregnancy: A transient state of epigenetic change favoring hypomethylation. *Epigenetics* **7**, 729–734 (2012).
24. Gaudet, M. M. *et al.* Associations of parity and age at first pregnancy with overall and cause-specific mortality in the Cancer Prevention Study II. *Fertility and Sterility* **107**, 179-188.e6 (2017).
25. Guo, H. *et al.* Relationship between biological ageing, premature mortality, life expectancy, and number of live births: a prospective cohort study of the UK women. *BMC Public Health* **25**, 3239 (2025).
26. Jin, C., Tooth, L. R., Xu, X. & Mishra, G. D. Do mothers or females without children have better health-related quality of life across their reproductive years? *Qual Life Res* **32**, 1481–1491 (2023).
27. Orchard, E. R., Rutherford, H. J. V., Holmes, A. J. & Jamadar, S. D. Matrescence: lifetime impact of motherhood on cognition and the brain. *Trends in Cognitive Sciences* S1364661322003023 (2023) doi:10.1016/j.tics.2022.12.002.
28. Orchard, E. R. & Rutherford, H. J. V. Birthing a new maternal cognition literature: 10 recommendations for future research. *Arch Womens Ment Health* https://doi.org/10.1007/s00737-024-01514-5 (2024) doi:10.1007/s00737-024-01514-5.
29. Shen, X. *et al.* Nonlinear dynamics of multi-omics profiles during human aging. *Nat Aging* **4**, 1619–1634 (2024).
30. Brunton, P. J. & Russell, J. A. The expectant brain: Adapting for motherhood. *Nature Reviews Neuroscience* **9**, 11–25 (2008).
31. Meyer, D. H. & Schumacher, B. Aging clocks based on accumulating stochastic variation. *Nat Aging* **4**, 871–885 (2024).
32. Ryan, C. P. *et al.* Pregnancy is linked to faster epigenetic aging in young women. *Proc. Natl. Acad. Sci. U.S.A.* **121**, e2317290121 (2024).
33. Harville, E. W. *et al.* Reproductive history and blood cell DNA methylation later in life: the Young Finns Study. *Clinical Epigenetics* **13**, 227 (2021).

34. Picci, G. *et al.* Dense sampling for mapping pituitary growth dynamics before, during, and after pregnancy. *J Neuroendocrinology* **38**, e70141 (2026).

35. Thrush, K. L., Higgins-Chen, A. T., Liu, Z. & Levine, M. E. R methylCIPHER: A Methylation Clock Investigational Package for Hypothesis-Driven Evaluation & Research. Preprint at https://doi.org/10.1101/2022.07.13.499978 (2022).

36. Clausen, A. N. *et al.* Assessment of brain age in posttraumatic stress disorder: Findings from the ENIGMA PTSD and brain age working groups. *Brain and Behavior* **12**, e2413 (2022).

37. Leonardsen, E. H. *et al.* Deep neural networks learn general and clinically relevant representations of the ageing brain. *NeuroImage* **256**, 119210 (2022).

38. Bashyam, V. M. *et al.* MRI signatures of brain age and disease over the lifespan based on a deep brain network and 14 468 individuals worldwide. *Brain* **143**, 2312–2324 (2020).

39. Kalc, P., Dahnke, R., Hoffstaedter, F., Gaser, C., & Alzheimer's Disease Neuroimaging Initiative. BrainAGE : Revisited and reframed machine learning workflow. *Human Brain Mapping* **45**, e26632 (2024).

40. Marcus, D. S. *et al.* Open Access Series of Imaging Studies (OASIS): Cross-sectional MRI Data in Young, Middle Aged, Nondemented, and Demented Older Adults. *Journal of Cognitive Neuroscience* **19**, 1498–1507 (2007).

41. LaMontagne, P. J. *et al.* OASIS-3: Longitudinal Neuroimaging, Clinical, and Cognitive Dataset for Normal Aging and Alzheimer Disease. Preprint at https://doi.org/10.1101/2019.12.13.19014902 (2019).

42. Taylor, J. R. *et al.* The Cambridge Centre for Ageing and Neuroscience (Cam-CAN) data repository: Structural and functional MRI, MEG, and cognitive data from a cross-sectional adult lifespan sample. *NeuroImage* **144**, 262–269 (2017).

43. Cam-CAN *et al.* The Cambridge Centre for Ageing and Neuroscience (Cam-CAN) study protocol: a cross-sectional, lifespan, multidisciplinary examination of healthy cognitive ageing. *BMC Neurol* **14**, 204 (2014).

44. Nooner, K. B. *et al.* The NKI-Rockland Sample: A Model for Accelerating the Pace of Discovery Science in Psychiatry. *Front. Neurosci.* **6**, (2012).

45. Gaser, C. *et al.* CAT: a computational anatomy toolbox for the analysis of structural MRI data. *GigaScience* **13**, giae049 (2024).

46. Pedersen, E. J., Miller, D. L., Simpson, G. L. & Ross, N. Hierarchical generalized additive models in ecology: an introduction with mgcv. *PeerJ* **7**, e6876 (2019).

47. Smith, S. M., Vidaurre, D., Alfaro-Almagro, F., Nichols, T. E. & Miller, K. L. Estimation of brain age delta from brain imaging. *NeuroImage* **200**, 528–539 (2019).

48. Butler, E. R. *et al.* Pitfalls in brain age analyses. *Human Brain Mapping* **42**, 4092–4101 (2021).

49. Benjamini, Y. & Hochberg, Y. Controlling the False Discovery Rate: A Practical and Powerful Approach to Multiple Testing. *Journal of the Royal Statistical Society: Series B (Methodological)* **57**, 289–300 (1995).
